# Machine learning–driven prediction of in-hospital cardiac arrest among critically ill patients with traumatic spinal cord injury: a multicenter cohort study

**DOI:** 10.3389/fcvm.2026.1818433

**Published:** 2026-09-14

**Authors:** Yixi Wang, Xinliang Peng, Wenzhe Li, Yang Wang, Xiangdong Jiang, Baoqiang Xu, Pengfei Pan, Yang Xiao, Qiuyuan Huang, Yuqian Li

**Affiliations:** 1 Department of Minimally Invasive Spine and Precision Orthopedics, Orthopedic Center, The First Affiliated Hospital of Xinjiang Medical University, Urumqi, China; 2 Heart Center, The First Affiliated Hospital of Xinjiang Medical University, Urumqi, China; 3 Department of Critical Care Medicine, The First Affiliated Hospital of Xinjiang Medical University, Urumqi, China; 4 Department of Anesthesiology, People's Hospital of Xinjiang Uygur Autonomous Region, Urumqi, China; 5 Department of Surgery, The Third Affiliated Hospital of Xinjiang Medical University, Urumqi, China; 6 Department of Surgery, The Fifth Affiliated Hospital of Xinjiang Medical University, Urumqi, China; 7 Department of Critical Care Medicine, Chongqing University Three Gorges Hospital, Chongqing, China; 8 Department of Orthopedics, Jingzhou First People's Hospital, Jingzhou, China; 9 Department of Anesthesiology, The First Affiliated Hospital of Xinjiang Medical University, Urumqi, China

**Keywords:** cardiac arrest prevention, critical care, in-hospital cardiac arrest, machine learning, risk prediction, traumatic spinal cord injury

## Abstract

**Background:**

In-hospital cardiac arrest (IHCA) represents a devastating complication among critically ill patients with traumatic spinal cord injury (TSCI), driven by profound autonomic dysfunction and systemic physiological instability. Despite the high mortality associated with these events, early recognition remains challenging, and dedicated risk stratification tools tailored to this vulnerable population are lacking.

**Methods:**

Patients from the US-based MIMIC-IV 3.1 and eICU-CRD 2.0 databases were randomly divided 7:3 into training and internal validation sets. Predictors recorded within 24 h of ICU admission were selected exclusively in the training set using univariable screening, Boruta, and group lasso. Eleven base learners were optimized by fivefold cross-validation and integrated into an L2-penalized stacking ensemble using out-of-fold probabilities. After internal validation, the best-performing model was locked and evaluated unchanged in an independent six-center Chinese cohort to assess transportability across populations and clinical settings differing in ethnic composition, geography, and practice patterns. Performance was assessed in terms of discrimination (ROC-AUC and PR-AP), calibration (calibration-in-the-large, calibration slope, Brier score, and expected calibration error), classification metrics, and clinical utility through decision-curve and lift analyses, while model predictions were interpreted using SHAP values.

**Results:**

The development cohort included 996 patients with 57 IHCA events, and the external cohort included 558 patients with 51 events. The stacking model with ten predictors retained good discrimination across the training, internal validation, and external validation cohorts, with ROC-AUCs of 0.953, 0.938, and 0.894 and corresponding PR-APs of 0.848, 0.794, and 0.774, respectively. Calibration was acceptable across all cohorts, with Brier scores of 0.013–0.020 and expected calibration errors of 0.020–0.033, and the 95% CIs for the external calibration-in-the-large (0.541) and calibration slope (1.318) included their respective ideal values, supporting the decision not to undertake *post hoc* recalibration. Decision-curve and lift analyses indicated favorable clinical utility, while SHAP analysis identified integrated patterns of autonomic dysfunction, perfusion compromise, metabolic disturbance, and overall physiological burden as the main contributors to prediction.

**Conclusion:**

The stacking model maintained discrimination and calibration across US and Chinese cohorts and may support interpretable early IHCA risk stratification and anticipatory management, although prospective evaluation is required before clinical implementation.

## 1 Introduction

Traumatic spinal cord injury (TSCI), affecting 15–52.5 individuals per million annually, causes not only severe neurological impairment but also an acute disruption of the neural regulation of circulation (1). In the early phase after injury, particularly following cervical or high thoracic TSCI, disruption of supraspinal input to sympathetic preganglionic neurons in the intermediolateral cell columns leaves vagal efferent activity intact, creating an acute autonomic imbalance characterized by sympathetic withdrawal and relative parasympathetic predominance (2). The resulting loss of chronotropic and vasomotor support produces bradycardia, peripheral vasodilation, venous pooling, reduced cardiac preload, and hypotension, while blunting the baroreflex responses required to maintain perfusion during physiological stress (3). Under these conditions, otherwise manageable disturbances such as hypoxemia, airway manipulation or electrolyte and acid–base abnormalities can overwhelm the limited compensatory reserve, precipitating severe bradyarrhythmia, neurogenic shock, or in-hospital cardiac arrest (IHCA) (2).

The clinical importance of this acute vulnerability is amplified by a narrow therapeutic window and by care pathways that remain largely extrapolated from general cardiac arrest algorithms (4). Before cardiac arrest, atropine, vasopressors, and cardiac pacing may be used to treat bradycardia or hypotension, but these interventions do not re-establish sympathetic vascular control and are usually triggered by overt hemodynamic instability, after compensatory reserve has already begun to fail (5, 6). Once IHCA occurs, the predominance of nonshockable rhythms such as asystole and pulseless electrical activity renders defibrillation ineffective and restricts resuscitation largely to cardiopulmonary resuscitation, epinephrine, and correction of reversible causes (7). Although these measures may restore spontaneous circulation, none directly addresses the autonomic dysfunction that predisposed the patient to cardiac arrest, which may contribute to persistent instability and the high mortality observed in this population (8, 9). Moreover, restoration of spontaneous circulation does not reverse the effects of the preceding global hypoperfusion, which can compound the initial mechanical injury by aggravating secondary ischemic damage to metabolically vulnerable spinal cord tissue (10).

These therapeutic limitations are particularly consequential given the reported 16.0% incidence of IHCA in this population and the limited reversibility of injury once cardiovascular collapse has occurred (11). Effective prevention requires a shift from reactive resuscitation to anticipatory management, supported by robust early risk prediction that enables timely hemodynamic optimization before irreversible decompensation ensues (12). However, existing monitoring strategies remain largely threshold-based and are poorly suited to capture the complex, evolving autonomic instability that precedes IHCA (13, 14). To address this gap, this study developed and validated a machine learning (ML)-based stacking model using routinely available clinical data from the first 24 h of admission, trained in the MIMIC-IV (15) and eICU cohorts (16), and externally validated across multiple centers in China, with the aim of providing a generalizable early-warning system to support preemptive management in this high-risk population.

## 2 Materials and methods

### 2.1 Data sources

A machine-learning model for early prediction of IHCA in critically ill patients with TSCI was developed using the MIMIC-IV 3.1 and eICU-CRD 2.0 databases (development cohort), released by MIT under a waiver of informed consent (Certification No. 57264471). Its generalizability was validated using external cohorts (external validation cohort) from the First, Third, and Fifth Affiliated Hospitals of Xinjiang Medical University (Ethics No. K202404-45), as well as from the People's Hospital of Xinjiang Uygur Autonomous Region (KY2024052264), Chongqing University Three Gorges Hospital (SXYYWD2022-058), and Jingzhou First People's Hospital (YJ202376), as shown in Figure 1.

**Figure 1:**
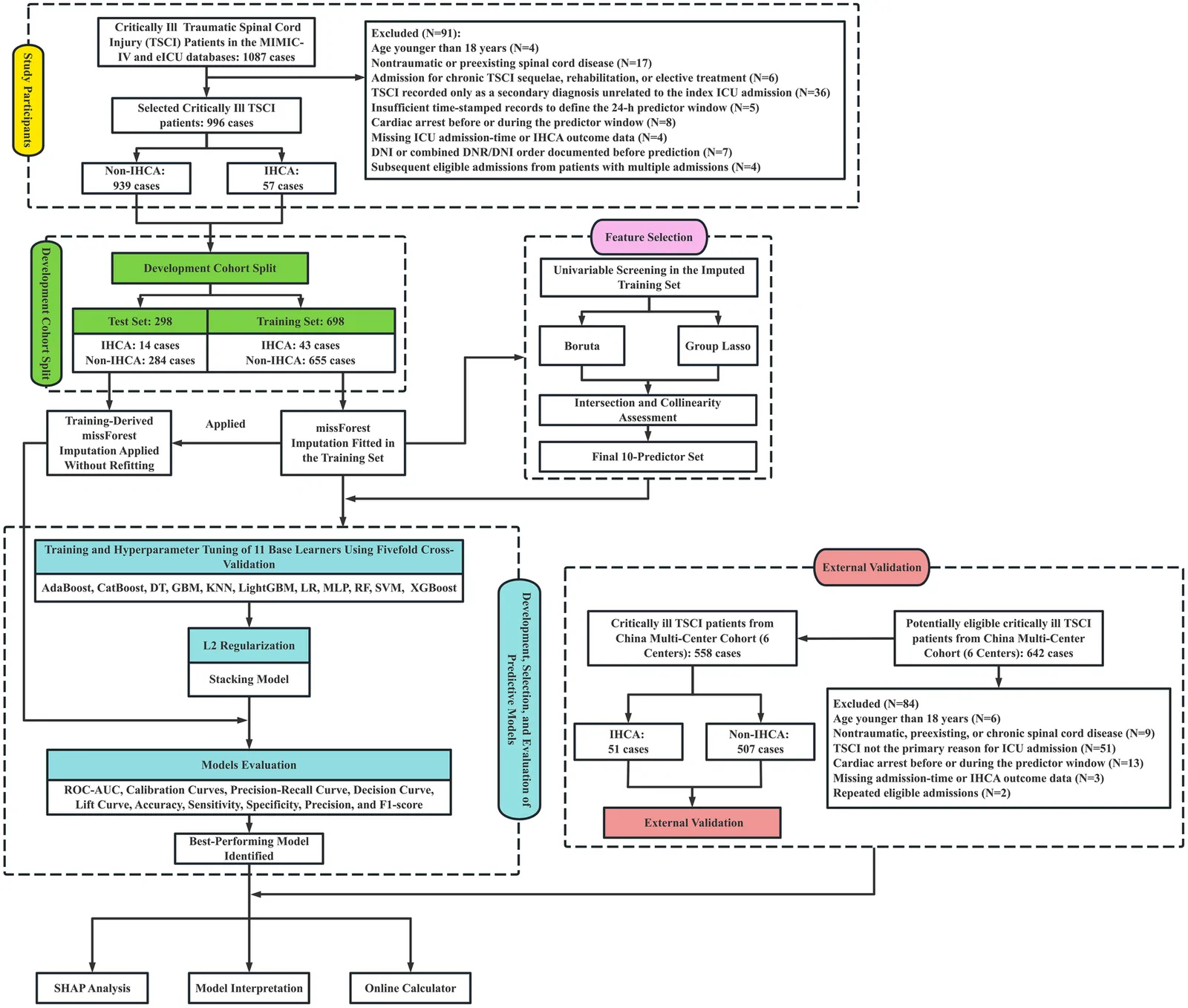
Study workflow for developing and validating a machine learning model to predict IHCA in critically ill TSCI patients. Potentially eligible TSCI-related ICU admissions were identified in MIMIC-IV and eICU-CRD (*n* = 1,087). After prespecified exclusions, 996 patients, including 57 with IHCA, formed the development cohort and were randomly divided into training (*n* = 698) and internal validation (*n* = 298) sets. A missForest imputation model was fitted in the training set and applied to the internal validation set without refitting. Within the imputed training set, predictors passing univariable screening were evaluated using Boruta and group lasso, followed by intersection and collinearity assessment to derive a fixed ten-predictor set. Eleven base learners were tuned using fivefold cross-validation, and their predictions were combined to construct an L2-regularized stacking ensemble as an additional candidate model. All candidate models were compared across the training and internal validation sets using discrimination, calibration, classification, and clinical utility metrics. The best-performing model was subsequently selected, locked, and applied without modification to an independent six-center Chinese cohort of 558 patients, including 51 with IHCA, selected from 642 potentially eligible admissions, to assess external performance and transportability across distinct populations and clinical settings. Following external validation, SHAP analysis was applied to the locked final model to quantify global feature importance and patient-specific contributions to predicted IHCA risk, after which the model was implemented as an online risk calculator.

### 2.2 Study population and data extraction

Patients with TSCI were initially identified in the MIMIC-IV 3.1 and eICU-CRD 2.0 databases using prespecified ICD-9-CM and ICD-10-CM codes listed in Supplementary Table 1. Inclusion criteria were: (1) age 18 years or older; (2) acute TSCI as the primary reason for ICU admission, requiring intensive treatment or close physiological monitoring; (3) availability of time-stamped ICU records spanning the first 24 h after admission to define the predictor window; and (4) availability of hospitalization records to determine IHCA status from the end of the predictor window through day 7 or until earlier discharge or death. Restricting outcome ascertainment to this period was intended to capture cardiac arrests associated with the acute consequences of TSCI and reduce confounding from later complications and treatment exposures. Exclusion criteria were: (1) nontraumatic or preexisting spinal cord disease; (2) admission for chronic TSCI sequelae, rehabilitation, or elective treatment; (3) TSCI recorded only as a secondary diagnosis unrelated to ICU admission; (4) cardiac arrest before or during the predictor window; (5) missing admission-time or outcome data; and (6) a Do Not Intubate (DNI) or combined Do Not Resuscitate (DNR)/DNI order documented before prediction. For patients with multiple eligible admissions, only the first TSCI-related ICU admission was retained. Isolated DNR orders did not preclude inclusion because the outcome was IHCA occurrence irrespective of whether resuscitation was attempted.

Candidate predictors comprised demographics, severity scores, vital signs, injury characteristics, laboratory measurements, treatments, and procedures recorded during the first 24 h of ICU admission. Variables available in structured fields were programmatically extracted from the MIMIC-IV and eICU-CRD tables using prespecified query scripts. Associated fracture, injury mechanism, and paralysis type, which were not consistently represented in structured fields, were ascertained from the original clinical records according to prespecified operational definitions documented in a standardized abstraction form (Supplementary File 1). The same form defined IHCA as the abrupt cessation of cardiac mechanical activity and specified explicit, time-stamped documentation of cardiac arrest or unequivocal equivalent terms, including cardiopulmonary arrest and cardiac standstill, as qualifying evidence. Wenzhe Li performed the initial abstraction of these predictors and IHCA outcomes, after which Yixi Wang verified each determination against the source records. Discrepancies were adjudicated by Yuqian Li through re-examination of the original clinical documentation, and the final verified or adjudicated determinations were used in subsequent analyses.

For external validation, a retrospective cohort was assembled from six hospitals across geographically distinct regions of China between January 2022 and January 2025 using the same eligibility criteria, predictor window, IHCA definition, and temporal restrictions as the development cohort. After the final predictor set had been locked solely on the basis of the training set, designated investigators at each center manually extracted predictor data and ascertained IHCA status from the original electronic medical records using the same variable definitions and outcome criteria as in the development cohort, after which Yixi Wang cross-checked the compiled data against the source records. Occasional discrepancies in predictor abstraction or IHCA ascertainment were adjudicated by Yuqian Li following re-evaluation of the source documentation. Formal DNR or DNI orders were not used at the participating Chinese centers during the study period, and no patients were excluded on this basis.

### 2.3 Data preprocessing and feature selection

Variables with more than 30% missingness in the pooled MIMIC-IV and eICU-CRD development cohort were excluded before the remaining data were randomly divided into a training set and an internal validation set in a 7:3 ratio. missForest was fitted using training-set predictors only, with the outcome excluded, and applied unchanged to the internal validation set. Within the imputed training set, variables intended solely for population description, such as race, or lacking prespecified clinical relevance, were excluded from model development regardless of univariable significance, after which candidate predictors passing univariable screening were refined using Boruta and group lasso, and their intersection was assessed for collinearity using Pearson correlation coefficients and variance inflation factors (VIFs). Collinear predictors (|r| > 0.80 or VIF >5) were reviewed iteratively in pairs, with retention based on stronger selection support, lower missingness, and greater clinical interpretability. Once locked, the final predictor set was applied unchanged to the internal validation set and external validation cohort. External predictor data were not imputed when complete, whereas any missing values were handled using the training-derived missForest procedure without refitting.

### 2.4 Handling class imbalance

Given the relatively low frequency of IHCA in the training set, synthetic oversampling was avoided because artificial observations could distort clinically meaningful associations in heterogeneous critical-care data. Class imbalance was instead addressed using algorithm-specific, cost-sensitive approaches while preserving the observed event distribution. Inverse-frequency class or sample weights were incorporated, where supported, into logistic regression (LR), decision tree (DT), multilayer perceptron (MLP), support vector machine (SVM), random forest (RF), adaptive boosting (AdaBoost), categorical boosting (CatBoost), gradient boosting machine (GBM), Light Gradient Boosting Machine (LightGBM), and Extreme Gradient Boosting (XGBoost). The boosting algorithms optimized weighted classification objectives, whereas RF combined class weighting with bootstrap aggregation. Because standard k-nearest neighbors (KNN) does not support cost-sensitive fitting, it was trained without model-specific imbalance correction, with its neighborhood and distance parameters tuned using fivefold cross-validation. KNN was retained as a non-cost-sensitive benchmark and as a potential source of complementary predictions for ensemble construction. The stacking model integrated out-of-fold (OOF) predictions from all 11 base learners through an L2-regularized meta-learner, combining models with different inductive structures while constraining ensemble complexity.

### 2.5 Statistical analysis and model development

Categorical variables were summarized as counts and percentages, and continuous variables as medians with interquartile ranges (IQRs). Baseline characteristics in the development cohort were compared by IHCA status using the *χ*² or Fisher's exact test for categorical variables and the Wilcoxon rank-sum test for continuous variables. These full-cohort comparisons were undertaken solely for descriptive reporting and were distinct from univariable predictor screening, which was performed exclusively in the training set. To characterize differences relevant to model transportability, basic cohort characteristics and the final selected predictors available in both the development and external validation cohorts were compared using the same statistical tests and standardized mean differences (SMDs), with an absolute SMD greater than 0.25 indicating meaningful imbalance (17).

Using the fixed predictors defined in Section 2.3, algorithm-specific hyperparameters for the 11 base learners were optimized by fivefold cross-validation within the training set. After the selected hyperparameters had been locked, each base learner generated out-of-fold probabilities, which were used both to evaluate its standalone training performance and, together with the observed IHCA outcomes, to train an L2-penalized logistic meta-learner. Each optimized base learner was subsequently refitted on the complete training set and applied independently to the internal validation set, where its probabilities were evaluated for standalone performance and entered into the locked meta-learner to generate stacking predictions. Across the training and internal validation sets, discrimination was assessed using the area under the receiver operating characteristic curve (ROC-AUC) and precision–recall average precision (PR-AP), whereas calibration was evaluated using calibration plots, calibration-in-the-large (CITL), calibration slope, Brier score, and expected calibration error (ECE), with 95% confidence intervals reported for all numerical performance estimates. Predicted probabilities were dichotomized at the threshold that maximized the F1 score in the training set and applied unchanged to the internal validation set and the external validation cohort to calculate accuracy, sensitivity, specificity, precision, and F1 score, while decision-curve analysis quantified net clinical benefit across clinically relevant thresholds and lift analysis assessed the concentration of IHCA events among patients assigned higher predicted risks. Among all evaluated candidate models (the 11 base learners and the Stacking ensemble), the model showing the most balanced and consistent discrimination, calibration, classification performance, and clinical utility across both the training and internal validation sets was selected and applied unchanged to the external validation cohort to assess transportability using the same performance framework. To contextualize its external performance and assess incremental predictive value, the selected model was additionally benchmarked against APACHE II, SOFA, and the training-derived LR model for ROC-AUC, PR-AP, and classification performance, with Acute Physiology and Chronic Health Evaluation II (APACHE II) and Sequential Organ Failure Assessment (SOFA) derived using harmonized 24-h definitions and the LR model applied without refitting. Model development and reporting followed the TRIPOD + AI guidelines (18), with Shapley additive explanations (SHAP) applied after external validation to provide global, individual-level, and injury-level subgroup interpretations of model predictions, followed by implementation of the validated model as an online risk calculator.

Statistical analyses, data imputation, and variable selection were conducted in R 4.4.3, and machine learning model development and evaluation were performed in Python 3.11. Two-sided *p*-values <0.05 were considered statistically significant.

## 3 Results

### 3.1 Study population and baseline characteristics

The development cohort comprised 996 critically ill patients with TSCI, including 57 who experienced IHCA, with baseline characteristics and comparisons by IHCA status presented in Table 1, whereas corresponding analyses restricted to the training set of 698 patients and 43 IHCA events are provided in Supplementary Table 2. The external validation cohort comprised 558 patients, including 51 with IHCA, and its baseline characteristics are reported in Supplementary Table 3. Because all baseline characteristics and prespecified predictors were routinely documented during standard ICU care, complete data were available for all external validation patients without imputation. Differences in baseline characteristics and final predictor distributions between the development and external validation cohorts are reported in Supplementary Table 4, with substantial between-cohort imbalances observed primarily for Mean Arterial Pressure (MAP) and potassium.

**Table 1:** Demographic data and baseline characteristics of the development cohort.

Variable	Non-IHCA Group (*N* = 939)	IHCA Group (*N* = 57)	*P*-value
Age [yr, median (IQR)]	58.00 (30.00)	65.00 (25.00)	0.052
Height [cm, median (IQR)]	175.00 (14.75)	175.00 (15.20)	0.998
Weight [kg, median (IQR)]	81.60 (26.99)	83.00 (15.00)	0.556
Gender [N (%)]			
Male	672 (71.6%)	44 (77.2%)	0.444
Female	267 (28.4%)	13 (22.8%)	
Race [N (%)]			
White	632 (67.3%)	32 (56.1%)	0.153
Black	109 (11.6%)	7 (12.3%)	
Other	198 (21.1%)	18 (31.6%)	
Rating System [median (IQR)]			
CCI	2.00 (4.00)	3.00 (2.00)	0.056
GCS	14.00 (4.00)	10.00 (2.00)	0.003*
SOFA	2.00 (4.00)	7.00 (5.00)	<0.001*
APACHE II	14.00 (11.00)	25.00 (13.00)	<0.001*
Spinal Injury Level [N (%)]			
Upper Cervical	177 (18.8%)	42 (73.7%)	<0.001*
Lower Cervical	145 (15.4%)	8 (14.0%)	
Cervical (Unspecified)	158 (16.8%)	1 (1.8%)	
Thoracic	141 (15.1%)	0 (0%)	
Lumbar	55 (5.9%)	0 (0%)	
Uncertain	263 (28.0%)	6 (10.5%)	
Associated Fracture [N (%)]			
Fracture	398 (42.4%)	30 (52.6%)	0.129
Non-Fracture	541 (57.6%)	27 (47.4%)	
Injury Mechanism [N (%)]			
Fall (High)	247 (26.4%)	20 (35.1%)	<0.001*
Fall (Low)	139 (14.8%)	5 (8.8%)	
Motor Vehicle	146 (15.5%)	22 (38.6%)	
Violence	222 (23.6%)	2 (3.5%)	
Unknown	185 (19.7%)	8 (14.0%)	
Paralysis Type [N (%)]			
Quadriplegia	126 (13.4%)	8 (14.0%)	0.021*
Paraplegia	114 (12.1%)	14 (24.6%)	
None/Uncertain	699 (74.5%)	35 (61.4%)	
Vasopressor [N (%)]			
Non-Use	729 (77.6%)	25 (43.9%)	<0.001*
Use	210 (22.4%)	32 (56.1%)	
Mechanical Ventilation [N (%)]			
Non-Use	682 (72.6%)	22 (38.6%)	<0.001*
Use	257 (27.4%)	35 (61.4%)	
Vital Signs [median (IQR)]			
SBP (mmHg)	125.00 (21.00)	118.00 (22.00)	0.001*
DBP (mmHg)	67.00 (13.00)	62.00 (11.00)	<0.001*
MAP (mmHg)	86.00 (14.00)	81.00 (12.00)	<0.001*
Heart Rate (beats/min)	63.00 (18.00)	52.00 (23.00)	<0.001*
Resp Rate (breaths/min)	17.00 (4.00)	19.00 (4.00)	0.001*
Temperature (°C)	36.30 (2.32)	35.40 (0.66)	<0.001*
Blood Gas [median (IQR)]			
SpO_2_ (%)	92.00 (6.00)	90.00 (12.00)	0.008*
Bicarbonate (mmol/L)	22.00 (4.00)	19.00 (5.00)	<0.001*
Laboratory Tests [median (IQR)]			
Hematocrit (%)	38.00 (7.30)	37.40 (10.10)	0.716
Hemoglobin (g/dL)	10.40 (3.50)	10.10 (2.80)	0.656
Platelets (10^9^/L)	172.00 (93.00)	159.00 (78.00)	0.106
WBC (10^9^/L)	13.70 (7.80)	16.20 (8.40)	0.014*
Calcium (mmol/L)	8.00 (1.10)	7.70 (0.70)	0.001*
Chloride (mmol/L)	107.00 (7.00)	109.00 (9.00)	0.002*
Potassium (mmol/L)	3.70 (0.60)	3.00 (0.90)	<0.001*
Sodium (mmol/L)	141.00 (4.50)	143.00 (6.00)	0.012
Glucose (mg/dL)	154.00 (70.00)	173.00 (97.00)	0.006*
Creatinine (mg/dL)	0.90 (0.44)	1.10 (0.70)	0.008*
BUN (mg/dL)	19.00 (13.00)	20.00 (14.00)	0.215

*means significant; For categorical variables with more than two levels, *P* values represent overall omnibus comparisons across all categories.

### 3.2 Feature selection and validation

Among variables associated with IHCA in univariable screening of the training set, Boruta and group lasso retained 12 and 13 predictors, respectively (Figures 2a,b), with their intersection comprising ten predictors, including Spinal Injury Level, Paralysis Type, Injury Mechanism, Vasopressor Use (VP), APACHE II, Serum Bicarbonate (HCO₃⁻), Serum Sodium (Na⁺), Serum Potassium (K⁺), MAP, and Body Temperature (Temp) (Figure 2c). Pearson correlation coefficients were uniformly low (Figure 2d), and all VIFs remained below the prespecified threshold (Figure 2e), indicating no problematic multicollinearity and supporting retention of all ten predictors for model development.

**Figure 2:**
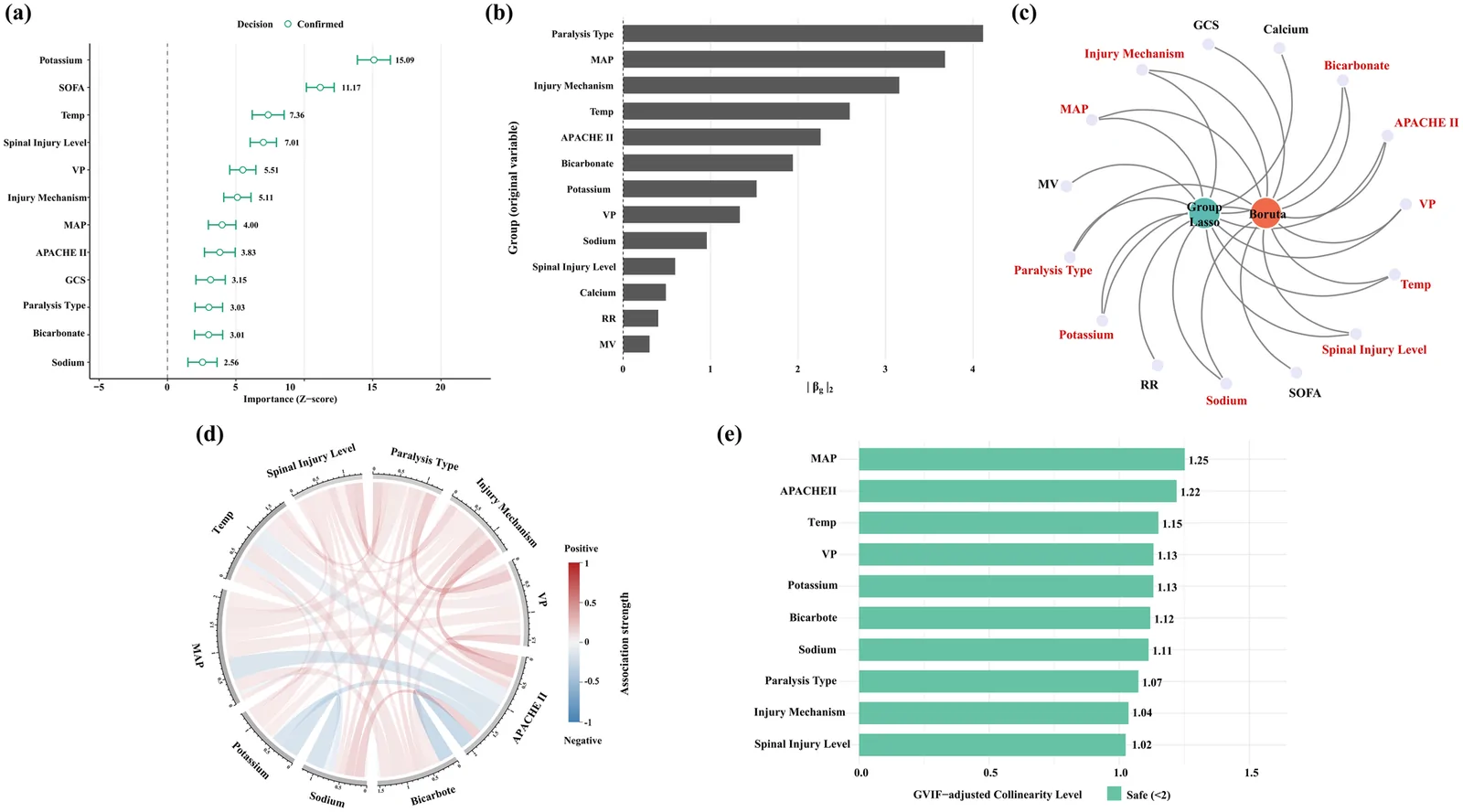
Feature selection and multicollinearity analysis. **(a)** Feature importance ranking from the Boruta algorithm based on Z-score statistics. **(b)** Standardized coefficient magnitudes (|*β*|) from group-LASSO feature selection. **(c)** Overlap between Boruta and group-LASSO selections identifying consensus predictors. **(d)** Correlation chord diagram of selected variables, with red and blue links indicating positive and negative associations. **(e)** GVIF-adjusted collinearity assessment showing all predictors within acceptable limits (<2).

### 3.3 Comparative performance of different models

In the training set, the stacking model showed the best overall performance, achieving the highest ROC-AUC (0.953) and PR-AP (0.848) and outperforming all first-level models (Figures 3a,b). After bootstrap correction for optimism, its performance remained relatively stable (ROC-AUC, 0.934; PR-AP, 0.823), suggesting a relatively limited degree of overfitting (Supplementary Table 5). Calibration curves and quantitative metrics (Figure 3c; Supplementary Table 6) indicated the closest agreement with the reference line, whereas individual models systematically underestimated IHCA risk. The stacking model also provided the greatest standardized net benefit (Figure 3d) and the highest enrichment, with lift values of 9.54×, 4.77×, and 3.26× in the top 10%, 20%, and 30% of predicted risk strata (Figure 3e). Performance across accuracy, precision, specificity, sensitivity, and F1-score was likewise most balanced for the stacking model, with declines among first-level learners in at least one or more metric (Figure 3f). Hyperparameter settings are shown in Supplementary Table 7.

**Figure 3:**
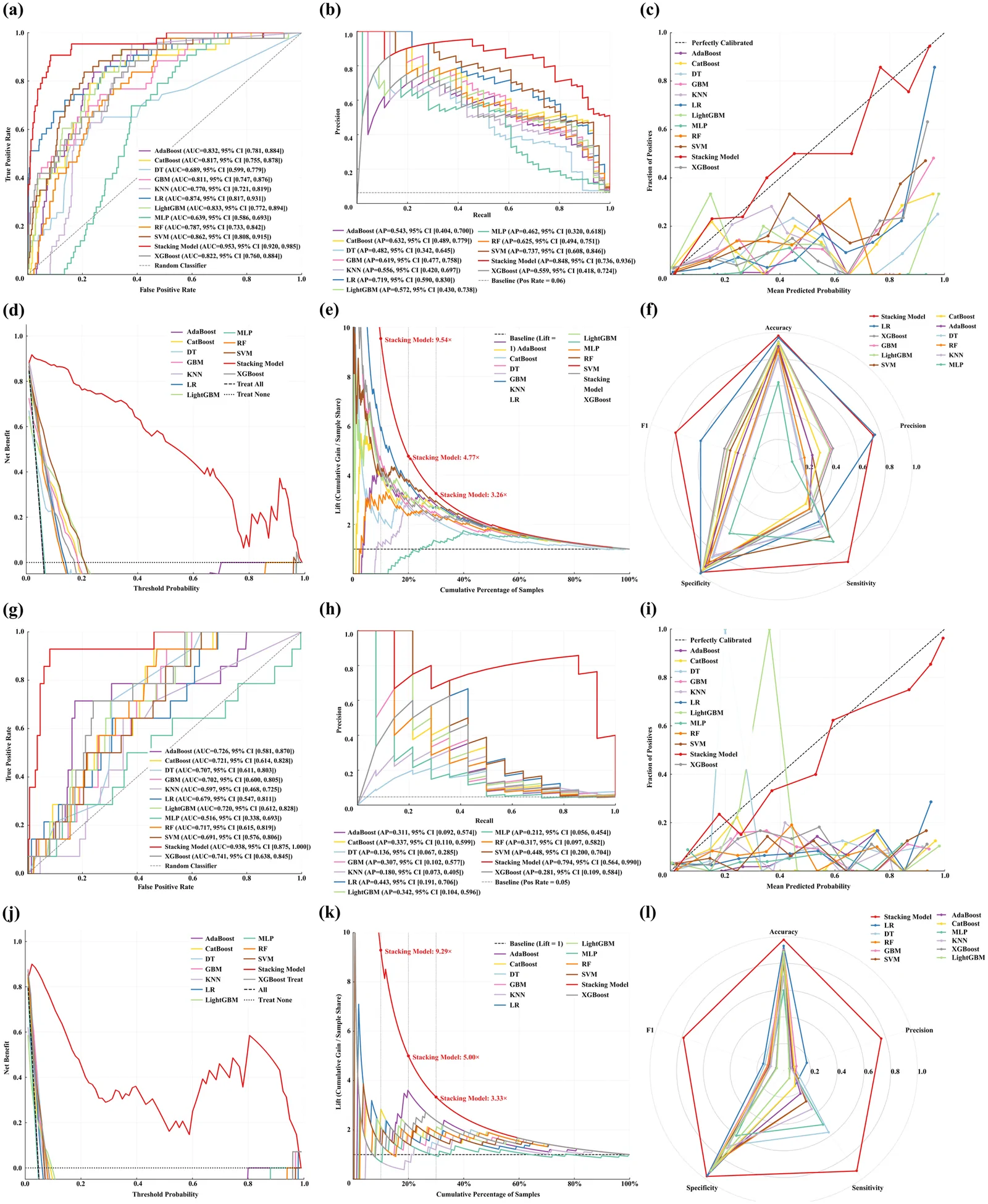
Comparative performance of machine learning models in the training and internal validation sets. (**a-f**) Training set results; (**g-l**) Internal validation set results. **(a,g)** ROC curves with AUC. **(b,h)** Precision–recall curves with average precision. **(c,i)** Calibration curves. **(d,j)** Decision curve analysis. **(e,k)** Lift curves. **(f,l)** Radar charts summarizing accuracy, precision, sensitivity, specificity, and F1-score. The stacking ensemble model consistently shows superior overall performance across discrimination, calibration, and clinical utility metrics.

In the internal validation set, the stacking model remained the top performer, retaining clear superiority across discrimination, calibration, and clinical utility. Its ROC-AUC (0.938) and PR-AP (0.794) declined only modestly from the OOF results and continued to exceed all first-level models (Figures 3g,h). The calibration curve of the stacking model remained closest to the ideal 45° reference line, whereas competing models consistently underestimated risk and exhibited poorer calibration stability (Figure 3i; Supplementary Table 2). The stacking model also preserved the highest net benefit across threshold probabilities and strong enrichment of true positives, achieving lift values of 9.29×, 5.00×, and 3.33× in the top 10%, 20%, and 30% risk strata, respectively (Figures 3j,k). Despite a narrower spread of all model performance than in the OOF set, none approached the stacking model's balanced profile across accuracy, precision, sensitivity, specificity, and F1-score (Figure 3l).

### 3.4 External validation

In the external cohort, the stacking model maintained strong performance, preserving discrimination, calibration, and clinical utility. ROC (Figure 4a) and PR (Figure 4b) analyses confirmed sustained discriminative ability, while calibration curves showed close agreement between predicted and observed risks with only minor deviations at extreme probabilities (Figure 4c; Supplementary Table 6). Decision curve analysis demonstrated consistent net benefit across a wide range of threshold probabilities (Figure 4d). The lift curve indicated effective risk stratification, with clear enrichment of true events among the highest-risk patients (Figure 4e), and radar analysis showed balanced performance across key metrics (Figure 4f).

**Figure 4:**
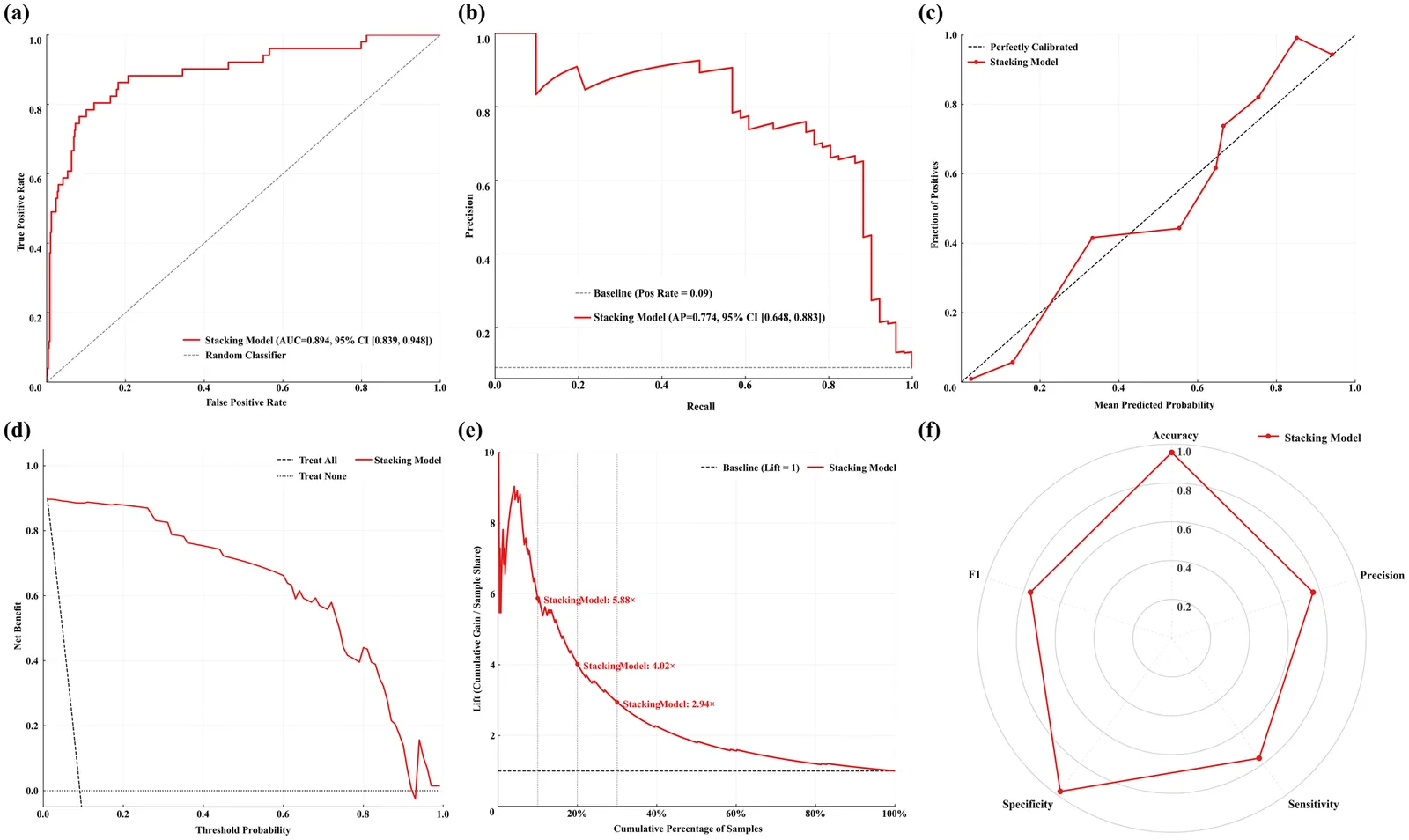
External validation performance of the stacking model. (**a**) ROC curve. (**b**) Precision–recall curve. (**c**) Calibration curve. (**d**) Decision curve analysis. (**e**) Lift curve. (**f**) Radar chart of classification metrics.

Benchmarking against the prespecified reference approaches showed that the stacking model provided the strongest discrimination and precision–recall performance and the most balanced classification profile in the external cohort. It identified more IHCA events with fewer false negatives while maintaining high specificity, whereas LR prioritized specificity at the expense of sensitivity, the APACHE II–based model showed the weakest event detection and F1 performance, and the SOFA-based model generated substantially more false positives (Table 2).

**Table 2:** Comparative performance of the stacking model and prespecified reference approaches in the external validation cohort.

Performance measure	Stacking model	Conventional multivariable LR	APACHE II–based model	SOFA-based model
ROC-AUC (95% CI)	0.894 (0.839–0.948)	0.857 (0.783–0.922)	0.706 (0.644–0.766)	0.800 (0.735–0.860)
PR-AP (95% CI)	0.774 (0.648–0.883)	0.675 (0.557–0.787)	0.176 (0.133–0.252)	0.325 (0.233–0.443)
TP	39	15	8	29
FN	12	36	43	22
FP	12	0	34	75
TN	495	507	473	432
Sensitivity	0.765	0.294	0.157	0.569
Specificity	0.976	1.000	0.933	0.852
PPV	0.765	1.000	0.190	0.279
NPV	0.976	0.934	0.917	0.952
Precision	0.765	1.000	0.190	0.279
Accuracy	0.957	0.935	0.862	0.826
F1	0.765	0.455	0.172	0.374

### 3.5 SHAP analysis

The SHAP analysis provided a global and individual-level view of how the model assembled IHCA risk. Contribution plots showed that predictions were chiefly influenced by potassium, APACHE II, bicarbonate, temperature, and MAP, while other factors functioned as secondary modifiers (Figure 5a). Summary SHAP patterns indicated that hypokalemia, greater systemic severity, metabolic acidosis, hypotension, and temperature abnormalities were associated with increased predicted risk, whereas preserved perfusion and metabolic stability exerted opposing effects (Figure 5b). The clustered heatmap further revealed that IHCA cases clustered in phenotypic patterns characterized by concurrent metabolic and hemodynamic disturbances rather than isolated abnormalities (Figure 5c). Case-level SHAP visualizations illustrated these additive effects in individual predictions (Survivor: Figure 5d; Death: Figure 5e).

**Figure 5:**
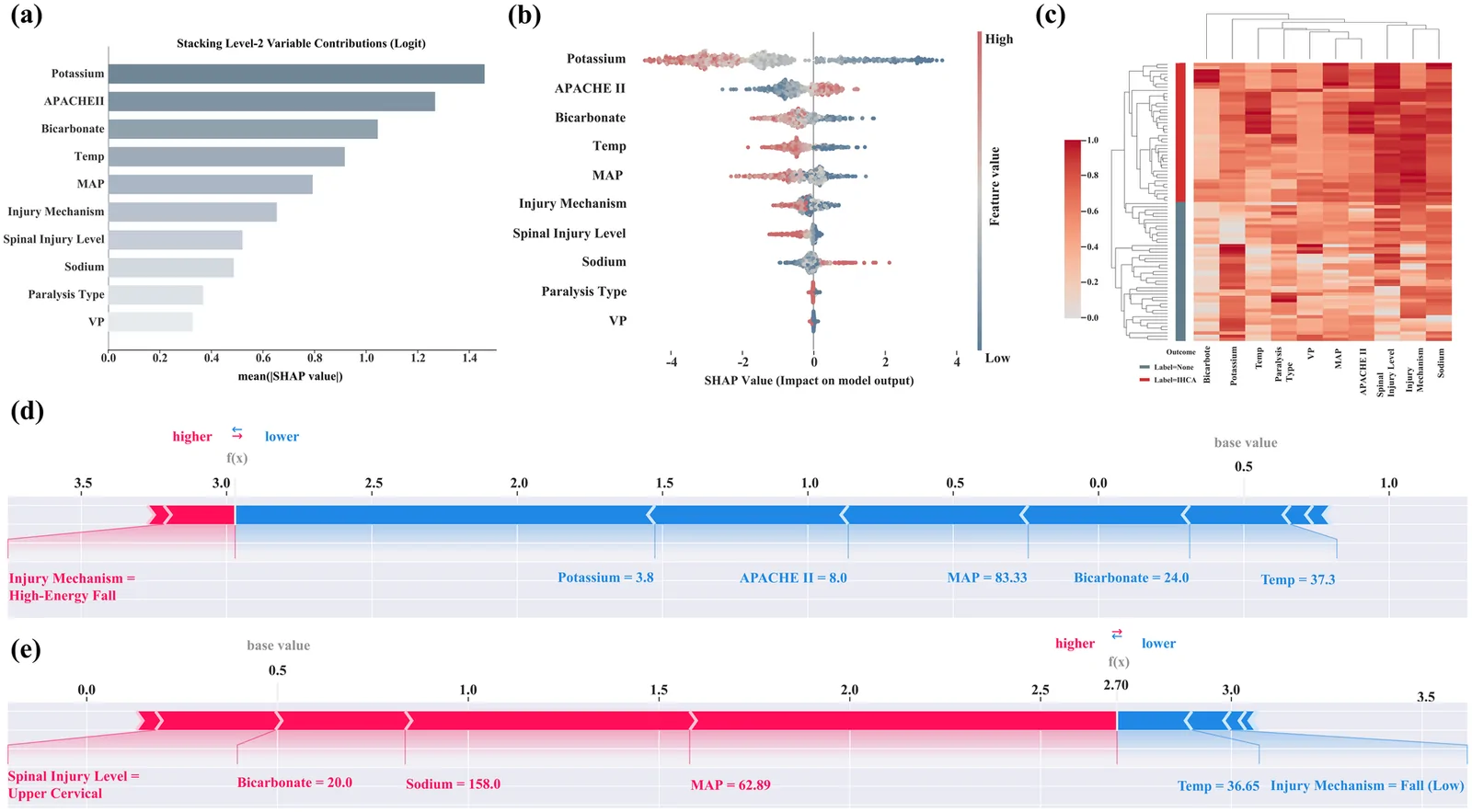
SHAP-based model interpretability analyses for the stacking model. **(a)** Global feature importance based on mean absolute SHAP values. **(b)** SHAP summary plot showing feature effects on model output. **(c)** SHAP heatmap with hierarchical clustering illustrating patient-level contribution patterns. **(d,e)** Representative individual SHAP force plots showing how features drive predictions toward higher (red) or lower (blue) IHCA risk relative to the base value.

To examine whether the model relied on different predictors across injury levels, SHAP values from the stacking model were summarized separately for patients with upper cervical injury, other documented injury levels comprising lower cervical, unspecified cervical, thoracic, and lumbar injuries, and uncertain injury level (Figures 6a–c). Potassium was consistently the leading contributor across all three subgroups, indicating a shared electrolyte-related component of IHCA risk estimation. Beyond this common signal, the hierarchy of predictor importance varied with injury presentation, with injury mechanism and APACHE II ranking next in upper cervical injury, APACHE II, temperature, and bicarbonate in other documented injury levels, and injury mechanism and paralysis type when anatomical localization was uncertain. These patterns suggest a layered predictive structure in which a common physiological foundation encompassing electrolyte disturbance, systemic severity, thermoregulatory instability, and metabolic status was modulated by injury-related information derived from injury mechanism and paralysis phenotype. The greater importance of paralysis type in the uncertain-level subgroup suggests that the model placed additional weight on the neurological phenotype documented during early assessment when precise anatomical localization was unavailable.

**Figure 6:**
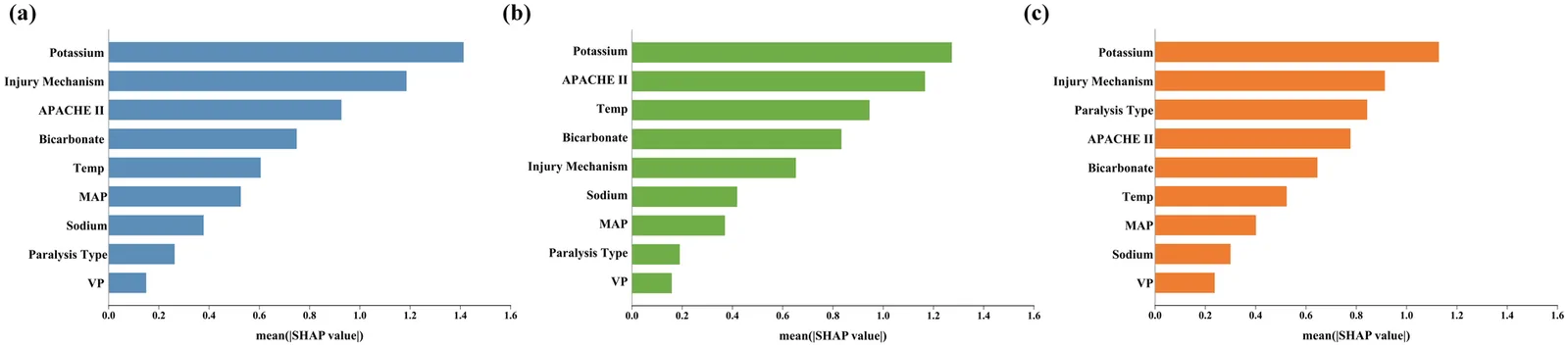
Injury-level subgroup analysis of predictor importance in the stacking model. Mean absolute Shapley additive explanation (SHAP) values were calculated in the training set and summarized separately for patients with **(a)** upper cervical injury, **(b)** other documented injury levels comprising lower cervical, unspecified cervical, thoracic, and lumbar injuries, and **(c)** uncertain injury level. Longer bars indicate a greater average contribution of the corresponding predictor to model output within each subgroup, irrespective of the direction of its effect.

### 3.6 Web calculator

An interactive online tool (Figure 7) based on the developed stacking framework has been deployed to facilitate real-time risk estimation and clinical support for IHCA among critically ill patients with TSCI (https://ml-ihca-prediction-ax9ixhlq2iqead6tdjdy7k.streamlit.app/).

**Figure 7:**
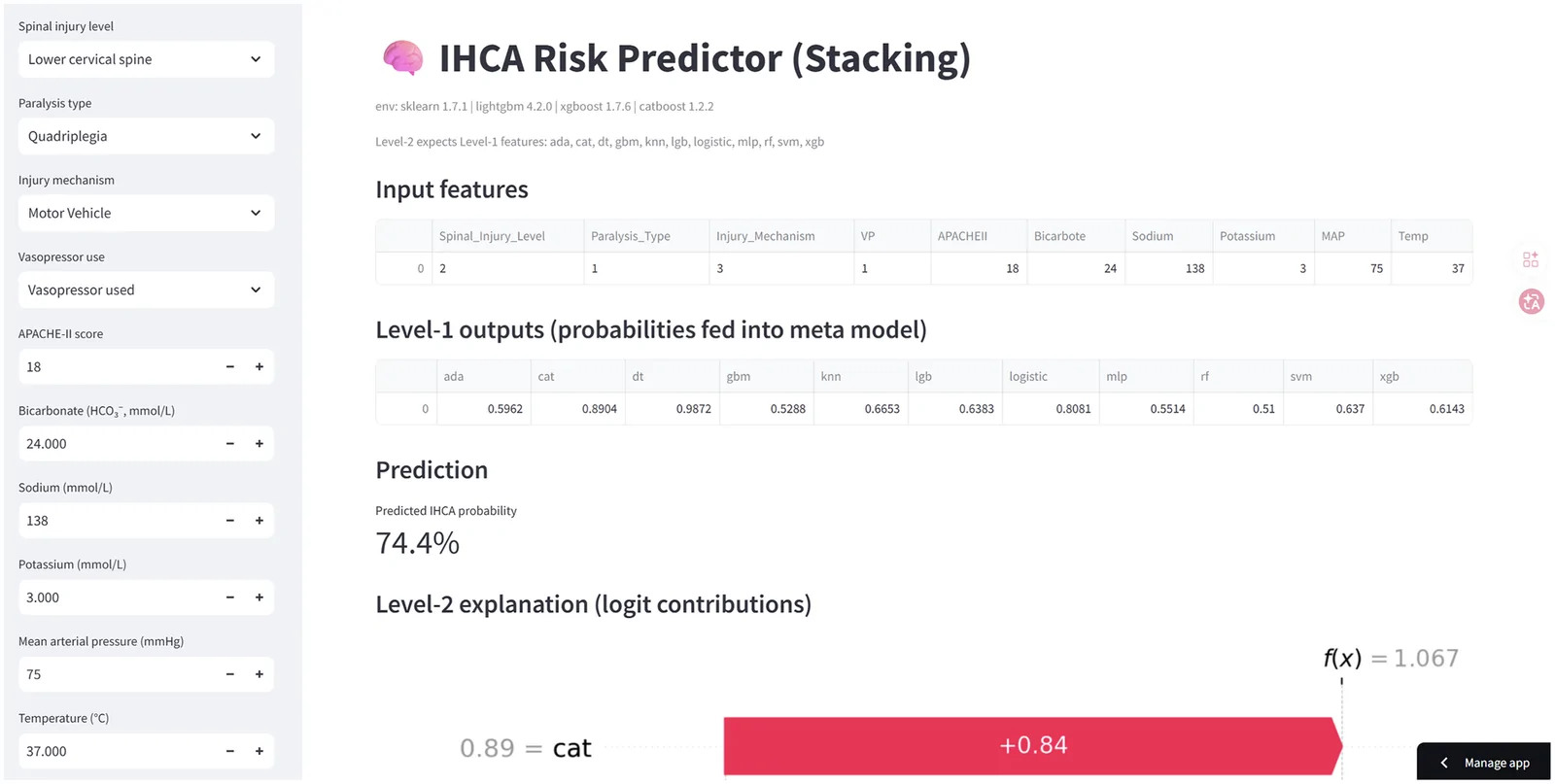
Online stacking-based IHCA risk calculator for patients with TSCI.

### 3.7 TRIPOD-AI reporting evaluation

Adherence to the TRIPOD-AI was systematically evaluated, demonstrating that the study met the majority of key reporting standards for AI-based prediction models, with the completed item-level checklist provided in Supplementary Material 2.

## 4 Discussion

This study provides the first TSCI-specific framework for early IHCA risk stratification using routinely available data from the first 24 h and shows consistent discrimination, acceptable calibration, and favorable clinical utility across development and external validation cohorts. The SHAP-derived structure further suggests that impending IHCA is represented not by any single severity measure but by a multidimensional phenotype integrating neurological injury, autonomic dysfunction, hemodynamic dependence, and metabolic disturbance, although translation of this latent vulnerability into clinical benefit will depend on whether the model can identify a manageable high-risk population sufficiently enriched for subsequent events to justify intensified surveillance without exacerbating alert fatigue.

Viewed from this perspective, the potential value of stacking may lie less in marginal refinements to individual risk ranking than in supporting the allocation of limited surveillance resources toward a clinically manageable group enriched for subsequent IHCA, in whom closer assessment and, where appropriate, clinical escalation may be justified (19). This distinction is consistent with earlier evaluations of eCART and DEWS (20, 21), which framed incremental value as improved event detection for a fixed false-alarm burden or fewer alarms for comparable event capture, and clarifies why the greater separation in PR-AP than in ROC-AUC is particularly relevant in this low-incidence setting (22). When applied unchanged to the external cohort, the locked stacking threshold captured approximately three-quarters of subsequent IHCA events within the one tenth of patients classified as high risk, yielding an eightfold enrichment over baseline incidence and a greater than 30-fold risk gradient between patients above and below the threshold. The benchmark approaches produced less favorable error trade-offs, with APACHE II and SOFA yielding both more false negatives and more false positives, whereas LR maintained perfect PPV only at the cost of missing most subsequent IHCA events. Compared with LR, stacking identified two additional IHCA events for every additional false-positive alert, suggesting that its gain in sensitivity reflected a more targeted expansion of the high-risk group rather than indiscriminate escalation. This distinction is particularly relevant after TSCI, because the synthesis by Kubo and Okawa suggests that, in severe cervical injury, seemingly routine interventions may precipitate IHCA when autonomic reserve is critically impaired, rendering early recognition of this vulnerability clinically actionable before generalized physiological deterioration becomes apparent (12). The practical value of stacking therefore lies not in a higher AUC as an abstract claim of superiority, but in identifying a clinically manageable high-risk group in whom rhythm and hemodynamic surveillance, correction of reversible metabolic disturbances, and precautions against autonomic triggers can be intensified without a disproportionate alert burden. Realizing this clinical potential requires not only reliable identification of high-risk patients but also a clinically intelligible account of the factors underlying their risk, making the anatomical, functional, and physiological significance of the SHAP-identified features integral to model interpretation in TSCI.

Among these features, injury level provided the key anatomical signal possibly because the lesion's rostrocaudal position reflected its proximity to cardiovascular regulatory regions, exemplified by cervical involvement, where disruption of descending sympathetic fibers could interrupt cardiac sympathetic projections and contribute to autonomic imbalance (23–25), while the indicator itself remained clinically accessible since the injured segment could generally be identified early through neurological findings and broad imaging patterns (26). Paralysis type further strengthened this framework by offering a clinically accessible potential indicator of the functional extent of tract interruption (27). Whereas the traditional complete–incomplete distinction was often obscured in the first 24 h by spinal shock, sedation, or limited patient cooperation (28, 29), the differentiation between quadriplegia and paraplegia could often be recognized earlier and might provide a pragmatic indication of the distribution of neurological impairment (30). This distinction also accorded with the greater predicted risk associated with tetraplegia in this study, a pattern consistent with the widespread loss of supraspinal sympathetic control observed in high cervical injuries (31). The injury mechanism contributed a biomechanical dimension by describing how traumatic energy might shape the spatial progression of cord damage (32). High-energy trauma may impart greater axial load and rotational shear, potentially producing multisegmental contusion and hemorrhagic extension that could disproportionately involve the continuous course of descending sympathetic fibers compared with the focal lesions typical of low-energy events, a pattern consistent with existing IHCA reports in TSCI (33, 34). Integrated, these indicators encoded complementary projections of a single physiological injury vector and may have provided information complementary to American Spinal Injury Association grading, which can be difficult to interpret early when transient somatic impairment obscures underlying pathway disruption (35). Clinically, the convergence of high cervical injury, quadriplegia, and a high-energy mechanism may warrant consideration of a high-acuity management posture involving continuous hemodynamic surveillance, strict avoidance of vagal triggers such as deep suctioning, vasopressor support, and readiness for pacing when otherwise clinically indicated (31).

Both lower MAP and early VP exposure emerged as leading predictors in SHAP analysis, a pattern that may plausibly reflect complementary markers of severe autonomic failure, with hypotension reflecting the abrupt loss of supraspinal sympathetic control and vasopressors mainly substituting for absent autonomic tone rather than causing hemodynamic harm (36). Early VP therefore likely marked patients whose circulation was already unstable, and within this narrow hemodynamic margin characteristic of autonomic failure, even small MAP shifts could further destabilize perfusion and promote systemic hypoperfusion or renewed instability (5, 37). At the cord level, falling MAP is expected to reduce spinal cord perfusion pressure and narrow the margin to ischemia, with consequent amplification of secondary injury through limited oxygen delivery and increased metabolic strain within the penumbra (38), while at the systemic level, the same decline risks breaching autoregulatory limits and compromising renal, splanchnic, and myocardial perfusion, thereby activating ischemic and inflammatory cascades that further destabilize an already compromised cardiovascular system (39, 40). However, excessive MAP elevation is not necessarily benign, as experimental T10 contusion–compression studies suggested that norepinephrine-mediated augmentation improved perfusion during compression yet heightened vulnerability to pressure-related vascular injury (41), and clinical observations after decompression similarly raised concerns regarding microvascular leakage and hemorrhagic expansion in reperfused tissue exposed to elevated pressures (42). Moreover, higher MAP targets often entail increased VP dosing, with consequent increases in afterload, myocardial oxygen demand, and arrhythmogenic stress, which aligned with reports of greater cardiovascular complications, particularly with dopamine-based strategies (43, 44). Taken together, MAP decline and early VP requirement appear to represent related manifestations of hemodynamic instability associated with autonomic dysfunction, with MAP acting as an integrative marker of global perfusion whose downward deviation can signal declining circulatory reserve and support individualized VP titration, including early norepinephrine when clinically indicated, cautious adjustment after decompression, continuous beat-to-beat monitoring, and readiness for pacing in selected patients, although whether such model-informed strategies reduce IHCA requires prospective evaluation (45).

SHAP analysis identified electrolyte disturbances as dominant biochemical predictors of IHCA after TSCI, with risk concentrated in patterns of low potassium, reduced bicarbonate, and elevated sodium. Although early TSCI can be accompanied by transient hyperkalemia through stress-activated potassium efflux during ischemia–reperfusion (46, 47), one possible interpretation is that the lower potassium observed in the IHCA cohort marked a transition beyond this initial efflux phase toward systemic processes favoring intracellular redistribution and renal loss, including catecholamine- and insulin-mediated shifts and renal potassium wasting (48, 49). The reduction in bicarbonate was consistent with a broader disturbance in acid–base homeostasis associated with spinal ischemia, hypoperfusion, lactate accumulation, and renal dysfunction (50). Contemporary studies have not demonstrated a consistent survival benefit from indiscriminate bicarbonate therapy in critical illness and have raised concerns regarding intracellular acidosis, impaired oxygen delivery, and worsening hypokalemia, suggesting that low bicarbonate should be interpreted primarily as a marker of the underlying metabolic disturbance rather than an isolated therapeutic target (51, 52). Within this evolving metabolic disturbance, disruption of autonomic control could also compromise systemic water–sodium regulation through impaired renal concentrating capacity and fluid balance, allowing free water loss to exceed sodium clearance and resulting in hypernatremia in the pre-arrest phase (53–55). In this context, increased serum sodium was more consistent with impaired neurohumoral and circulatory regulation than with an isolated electrolyte abnormality, and its reported association with poor neurological recovery further supports its interpretation as a marker of systemic decompensation (56). Taken together, these electrolyte disturbances may mark a biochemical inflection point at which the spinal cord, kidneys, and myocardium are progressively losing the capacity to maintain ionic and metabolic gradients, such that their convergence could signal a rapidly narrowing window to prevent brady-asystolic cardiac arrest. Management should therefore prioritize identifying and reversing upstream drivers, including unrecognized renal potassium loss, bicarbonate-related shifts, worsening acidosis, and progressive hyperosmolar water deficit, rather than relying on late correction of the electrolyte abnormalities themselves (57).

The APACHE II score measured within the first 24 h was incorporated into the model to capture the global physiological burden at presentation and the early erosion of systemic reserve that followed TSCI (58). Prior studies have shown that higher admission APACHE II scores are associated with long-term mortality in this population, even after adjustment for neurological impairment, with each one-point increment reportedly corresponding to an approximately 18% relative increase in mortality risk, underscoring the prognostic information conveyed by early physiological disequilibrium beyond anatomical injury severity (59, 60). Evidence from cardiac arrest populations further informs this interpretation, as the modest discrimination of admission APACHE II scores, with an AUC near 0.6 but increasing steadily as subsequent updated scores accumulated, suggests their greater sensitivity to latent vulnerability than to established multi-organ failure (61). This distinction was highly relevant to TSCI, where abrupt sympathetic disruption and declining ventilatory effectiveness could produce a profound yet initially understated destabilization of systemic reserve that potentially preceded overt organ dysfunction (62). Within this context, an elevated early APACHE II score may be viewed as a marker of diminished physiological resilience, leaving patients more susceptible to progression toward cardiac arrest in response to otherwise manageable changes in hemodynamic load, ventilatory demand, or inflammatory stress (63). Recognition of this state provides a rationale for a stability-focused preventive strategy emphasizing continuous monitoring, early ventilatory support, and vigilant management of infection and metabolic stress.

Besides the above factors, temperature was included in the model as an additional physiological variable, with the IHCA group showing a higher prevalence of hypothermia, a disturbance of particular concern in TSCI because it may reflect failure of hypothalamic–sympathetic thermoregulation, in which impaired vasoconstriction, shivering, and sweating allow heat loss to outpace heat production and produce a rapid, uncompensated decline in core temperature (63, 64). The association between hypothermia and IHCA in this cohort was consistent with this biologically plausible mechanism, since cardiac electrophysiology is highly temperature sensitive and even modest cooling slows sodium-channel kinetics, prolongs action-potential duration, depresses sinus-node automaticity, and amplifies vagal influence, effects normally counterbalanced by sympathetic activation but potentially left unopposed in high-level TSCI (65–67). Therefore, hypothermia may represent not only a marker of autonomic failure but also a contributor to hemodynamic deterioration, potentially promoting bradyarrhythmia and progression toward asystole through processes resembling hypothermic cardiac arrest but intensified by profound autonomic disconnection (68, 69). These findings do not establish that induced mild hypothermia is harmful, but they challenge the assumption that it is uniformly beneficial in acute TSCI. Controlled cooling can reduce metabolic demand within the spinal cord, whereas spontaneous hypothermia may denote advanced autonomic failure and a myocardium with limited repolarization reserve and minimal sympathetic buffering, creating a physiological state in which further cooling could be harmful (70). Clinically, temperature may therefore be regarded as a dynamic marker of autonomic integrity, with falling core values before overt cardiovascular instability providing a rationale for prompt reassessment of autonomic and hemodynamic reserve and careful reconsideration of ongoing cooling strategies.

This study applied an ML framework to the early prediction of IHCA after TSCI and demonstrated encouraging performance, although several limitations should be acknowledged. First, the training set contained 43 IHCA events for ten fixed predictors, corresponding to 4.3 events per predictor. Although below conventional heuristics for unpenalized regression, this ratio does not directly represent the effective complexity of regularized nonlinear ensembles. Overfitting was mitigated through predictor restriction, cross-validated hyperparameter optimization, out-of-fold construction of the stacking model, and L2 regularization, with modest attenuation after optimism correction and preserved performance in independent validation cohorts providing empirical support for model stability. Nevertheless, further validation in cohorts with more events remains warranted. Second, feature selection was performed once in the complete training set rather than repeated within each cross-validation fold, which may have introduced limited selection-related optimism into the training-set out-of-fold estimates. However, the predictor set was locked before validation and applied unchanged to the internal and external cohorts, neither of which contributed to imputation, feature selection, or model fitting. This issue therefore primarily concerns estimation of training performance and does not constitute direct information leakage into external validation, although fully nested resampling would provide a more stringent assessment in future studies. Third, differences in case mix and predictor distributions were observed between the US development cohort and the Chinese external cohort, probably reflecting variation in population characteristics, clinical practice, transfer pathways, and data-acquisition intensity. In the Chinese external cohort, patients commonly received initial stabilization in emergency or resuscitation units before ICU transfer, which may have modified the physiological and laboratory values subsequently recorded during the ICU predictor window. They may also reflect differences in measurement density, because repeated sampling in MIMIC-IV and eICU-CRD increased the likelihood of capturing physiological extremes, whereas external values were often derived from fewer measurements within the same 24-h window. Individual standardized mean differences therefore cannot be attributed solely to underlying population characteristics, but should be interpreted as the combined result of case mix, pre-ICU treatment, and data-acquisition practices. Nevertheless, the direction of predictor differences between patients with and without IHCA was broadly consistent across cohorts, supporting preservation of the underlying risk pattern. Data completeness was also achieved differently, as the development cohort required exclusion of highly incomplete variables and missForest imputation, whereas the external predictors were retrieved retrospectively and verified against source records. Because no external patient was excluded for predictor missingness and the locked variables were routinely documented in all eligible patients, this difference was unlikely to have materially altered external cohort composition. However, targeted retrospective retrieval may provide more complete information than would be immediately available during prospective implementation. Future validation using harmonized measurement schedules and real-time data collection is therefore needed to confirm both transportability and operational feasibility. Fourth, SHAP analysis identifies features contributing to model prediction but does not establish biological mechanisms or causal directionality. The concordance of the observed patterns with established TSCI physiology lends clinical plausibility to the model, while the proposed mechanistic explanations remain hypothesis-generating. This limitation concerns mechanistic interpretation rather than predictive performance and should be addressed through prospective studies incorporating temporally resolved physiological data. Finally, the model was deliberately developed for early IHCA prediction and was not intended to address long-term neurological outcomes or post-discharge complications. Detailed ventilatory parameters, inflammatory biomarkers, and fluid-balance measurements were not consistently available across cohorts and might provide additional predictive information. Future studies incorporating richer longitudinal data could further refine risk estimation and determine whether model-guided surveillance improves clinical outcomes.

## 5 Conclusion

IHCA in critically ill TSCI patients represents a manifestation of early autonomic and systemic instability that calls for anticipatory rather than reactive management, and this study developed and externally validated a stacking model based on routinely available early clinical data that demonstrated stable discrimination, acceptable calibration, and consistent clinical utility. Model interpretation indicated that risk estimation was driven by integrated patterns of autonomic dysfunction, perfusion compromise, metabolic disturbance, and overall physiological burden, suggesting that the model may assist early risk stratification and targeted monitoring in this high-risk population, while further prospective validation is required to determine its clinical impact.

## Data Availability

The datasets presented in this study can be found in online repositories. The names of the repository/repositories and accession number(s) can be found in the article/Supplementary Material.
