# Supplementary material for: Machine learning–driven prediction of in-hospital cardiac arrest among critically ill patients with traumatic spinal cord injury: a multicenter cohort study
Source: Front Cardiovasc Med. 2026 Sep 14;13:1818433. doi: 10.3389/fcvm.2026.1818433 (PMC13617107; doi:10.3389/fcvm.2026.1818433)
Supplement: Supplementary file 2 [file Supplementaryfile1.docx]

**Supplementary Material 1**

**Standardized Data Abstraction Form for the TSCI–IHCA Study**

**1. Purpose and general abstraction rules**

This form standardizes retrospective extraction of candidate predictors recorded during the first 24 h after ICU admission and ascertainment of in-hospital cardiac arrest (IHCA) outcomes in the development and external validation cohorts. One form should be completed for each first eligible TSCI-related ICU admission, using only study-assigned or deidentified identifiers.

| **Predictor window** | From ICU admission (time 0) through 24 h after admission. Measurements or treatments initiated after this window must not be used as predictors. |
| --- | --- |
| **Outcome window** | From completion of the 24-h predictor window through day 7 after ICU admission, or until earlier hospital discharge or death. |
| **Source records** | Use structured electronic medical record fields whenever available and narrative clinical documentation when the required information is absent or ambiguous in structured fields. |
| **Multiple measurements** | Apply the prespecified variable-specific 24-h aggregation rule used in the analysis dataset, record the selected value and its date/time, and do not select values with knowledge of subsequent IHCA status. |
| **Missing or uncertain data** | Do not infer undocumented values. Record “Not available” for missing numerical data and “Unclear” or “Uncertain” only where permitted by the operational definition. |
| **Source evidence** | For manually ascertained variables, record a concise source phrase together with the note type, date/time, and source locator; avoid direct patient identifiers. |

**2. Case identification and eligibility**

| **Field** | **Entry** | **Field** | **Entry** |
| --- | --- | --- | --- |
| Study case ID | ____________________________ | Cohort | [ ] Development [ ] External validation |
| Database or center | ____________________________ | Source encounter ID | ____________________________ |
| ICU admission date/time | ____ / ____ / ______ ____:____ | Predictor-window end | ____ / ____ / ______ ____:____ |
| Outcome-window end | ____ / ____ / ______ ____:____ | Reason window ended | [ ] Day 7 [ ] Discharge [ ] Death |
| Initial extractor | ____________________________ | Extraction date | ____ / ____ / ______ |
| Verifier | ____________________________ | Verification date | ____ / ____ / ______ |

**Eligibility checklist**

| **Criterion** | **Operational check** | **Assessment** |
| --- | --- | --- |
| Inclusion | Age 18 years or older | [ ] Yes [ ] No |
| Inclusion | Acute TSCI was the primary reason for ICU admission and required intensive treatment or close physiological monitoring | [ ] Yes [ ] No |
| Inclusion | Time-stamped ICU records adequately define the first 24-h predictor window | [ ] Yes [ ] No |
| Inclusion | Hospital records permit ascertainment of IHCA status through the prespecified outcome window | [ ] Yes [ ] No |
| Exclusion | Nontraumatic or preexisting spinal cord disease | [ ] Yes [ ] No |
| Exclusion | Admission for chronic TSCI sequelae, rehabilitation, or elective treatment | [ ] Yes [ ] No |
| Exclusion | TSCI recorded only as a secondary diagnosis unrelated to ICU admission | [ ] Yes [ ] No |
| Exclusion | Cardiac arrest before or during the 24-h predictor window | [ ] Yes [ ] No |
| Exclusion | Missing ICU admission time or outcome information | [ ] Yes [ ] No |
| Exclusion | DNI or combined DNR/DNI order documented before prediction | [ ] Yes [ ] No [ ] Not used |
| Final decision | First eligible TSCI-related ICU admission retained | [ ] Include [ ] Exclude |

**3. Candidate predictor abstraction**

The domains and categories below reproduce the variables reported in Table 1. Record the analytic value derived within the first 24 h, together with the supporting source and date/time where applicable.

| **Variable** | **Operational definition or allowed category** | **Unit or coding** | **Abstracted value / source** |
| --- | --- | --- | --- |
| **A. Demographics** | | | |
| Age | Age at index ICU admission | years | ____________________________ |
| Height | Height documented for the index admission | cm | ____________________________ |
| Weight | Weight documented for the index admission | kg | ____________________________ |
| Sex | Recorded sex | Female / Male / Other or unknown | ____________________________ |
| Race | Race category recorded in the source database or medical record | White / Black / Other or unknown | ____________________________ |
| **B. Severity scores** | | | |
| CCI | Charlson Comorbidity Index calculated using the prespecified study algorithm | score | ____________________________ |
| GCS | Glasgow Coma Scale within the predictor window according to the analysis rule | score | ____________________________ |
| SOFA | Sequential Organ Failure Assessment calculated from the first 24 h | score | ____________________________ |
| APACHE II | Acute Physiology and Chronic Health Evaluation II calculated from the first 24 h | score | ____________________________ |
| **C. TSCI characteristics** | | | |
| Spinal injury level | Most specific anatomical level documented within 24 h. Upper cervical=C1–C4; lower cervical=C5–C8; cervical unspecified; thoracic; lumbar; or uncertain | category | ____________________________ |
| Associated fracture | Acute vertebral or spinal fracture associated with the index TSCI and explicitly documented in imaging, diagnosis, operative, or clinical records | Yes / No / Unclear | ____________________________ |
| Injury mechanism | Mechanism responsible for the index injury | High-level fall / Low-level fall / Motor vehicle / Violence / Unknown | ____________________________ |
| Paralysis type | Neurological phenotype explicitly documented within 24 h; do not infer completeness from sedation, spinal shock, or an incomplete examination | Quadriplegia / Paraplegia / None or uncertain | ____________________________ |
| **D. Treatments and procedures during the first 24 h** | | | |
| Vasopressor | Any vasopressor administered for hemodynamic support during the predictor window | Non-use / Use | ____________________________ |
| Mechanical ventilation | Invasive mechanical ventilation delivered through an endotracheal or tracheostomy tube during the predictor window | Non-use / Use | ____________________________ |
| **E. Vital signs and blood gas measurements** | | | |
| SBP | Systolic blood pressure summarized using the prespecified 24-h analysis rule | mm Hg | ____________________________ |
| DBP | Diastolic blood pressure summarized using the prespecified 24-h analysis rule | mm Hg | ____________________________ |
| MAP | Mean arterial pressure summarized using the prespecified 24-h analysis rule | mm Hg | ____________________________ |
| Heart rate | Heart rate summarized using the prespecified 24-h analysis rule | beats/min | ____________________________ |
| Respiratory rate | Respiratory rate summarized using the prespecified 24-h analysis rule | breaths/min | ____________________________ |
| Temperature | Body temperature summarized using the prespecified 24-h analysis rule | °C | ____________________________ |
| SpO₂ | Peripheral oxygen saturation summarized using the prespecified 24-h analysis rule | % | ____________________________ |
| Bicarbonate | Serum bicarbonate summarized using the prespecified 24-h analysis rule | mmol/L | ____________________________ |
| **F. Laboratory measurements** | | | |
| Hematocrit | Hematocrit within the predictor window | % | ____________________________ |
| Hemoglobin | Hemoglobin within the predictor window | g/dL | ____________________________ |
| Platelets | Platelet count within the predictor window | 10⁹/L | ____________________________ |
| WBC | White blood cell count within the predictor window | 10⁹/L | ____________________________ |
| Calcium | Serum calcium within the predictor window | mmol/L | ____________________________ |
| Chloride | Serum chloride within the predictor window | mmol/L | ____________________________ |
| Potassium | Serum potassium within the predictor window | mmol/L | ____________________________ |
| Sodium | Serum sodium within the predictor window | mmol/L | ____________________________ |
| Glucose | Blood glucose within the predictor window | mg/dL | ____________________________ |
| Creatinine | Serum creatinine within the predictor window | mg/dL | ____________________________ |
| BUN | Blood urea nitrogen within the predictor window | mg/dL | ____________________________ |

**4. Manual ascertainment of TSCI characteristics**

| **Variable** | **Operational definition** | **Recorded determination and evidence** |
| --- | --- | --- |
| Associated fracture | Classify as Yes only when an acute vertebral or spinal fracture associated with the index TSCI is explicitly documented in a radiology report, diagnosis, operative note, or clinical assessment. Classify as No when adequate documentation indicates no associated fracture; use Unclear when the available record is insufficient. | Final category: ____________________ Source phrase and locator: ________________________________________________ |
| Injury mechanism | Assign the mechanism responsible for the index TSCI using the original source wording. High-level fall denotes a fall from an elevation; low-level fall denotes a same-level or low-height fall; motor vehicle includes vehicle-related collision; violence includes assault or penetrating interpersonal injury; use Unknown when no reliable mechanism is documented. | Final category: ____________________ Source phrase and locator: ________________________________________________ |
| Paralysis type | Use explicit neurological documentation within the first 24 h. Quadriplegia includes tetraplegia affecting all four limbs; paraplegia affects the lower limbs without upper-limb involvement. Use None/Uncertain when paralysis is not documented or cannot be determined reliably because of spinal shock, sedation, altered consciousness, or an incomplete examination. Do not infer complete versus incomplete injury from this field. | Final category: ____________________ Source phrase and locator: ________________________________________________ |

**5. IHCA outcome ascertainment**

**5.1 Operational definition**

IHCA was defined as the abrupt cessation of cardiac mechanical activity after ICU admission, evidenced clinically by the absence of a palpable central pulse or other signs of circulation. A qualifying study outcome required explicit, time-stamped documentation in the clinical record that an in-hospital cardiac arrest had occurred and had to fall after completion of the 24-h predictor window and no later than day 7 after ICU admission or the earlier time of hospital discharge or death.

**5.2 Qualifying documentation**

| **Primary qualifying evidence** | Explicit documentation of “cardiac arrest” or an unequivocal clinically equivalent term, including “cardiopulmonary arrest”, “cardiorespiratory arrest”, or “cardiac standstill”. “Asystolic arrest” and “pulseless electrical activity arrest” also qualify when the record clearly denotes loss of circulation rather than an isolated monitor finding. |
| --- | --- |
| **Required temporal information** | The event date/time must be explicitly recorded or reliably established from contemporaneous documentation and must lie within the prespecified outcome window. If the exact time cannot be established, classify the event as Unclear pending verification or adjudication. |
| **Acceptable source locations** | Physician progress or event notes, nursing records, code or resuscitation records, discharge or death summaries, and time-stamped diagnosis or clinical-event documentation. Record the source type, date/time, and locator. |
| **Supportive evidence** | Chest compressions or CPR, resuscitation-dose epinephrine, defibrillation, a code-blue activation, documented asystole or pulseless electrical activity, and return of spontaneous circulation may corroborate a documented arrest but must not independently define IHCA without explicit evidence that cardiac arrest occurred. |
| **Resuscitation not required** | IHCA classification is based on occurrence of cardiac arrest, not on whether resuscitation was attempted or successful. A documented event therefore remains eligible in a patient with an isolated DNR order. |
| **Multiple events** | If more than one qualifying IHCA occurs, record the first event within the outcome window as the study outcome and document subsequent events in the notes. |

**5.3 Findings that do not independently establish IHCA**

| **Check** | **Non-qualifying or insufficient evidence** |
| --- | --- |
| [ ] | Bradycardia, hypotension, syncope, transient loss of consciousness, or hemodynamic deterioration without explicit documentation of cardiac arrest. |
| [ ] | An isolated arrhythmia, telemetry label, monitor artifact, or rhythm description without clinical confirmation of loss of circulation. |
| [ ] | Respiratory arrest or apnea without documented cessation of cardiac mechanical activity. |
| [ ] | CPR, chest compressions, epinephrine administration, defibrillation, or code activation recorded without explicit or unequivocal documentation that cardiac arrest occurred. |
| [ ] | Death without documentation of a preceding cardiac arrest. |
| [ ] | Out-of-hospital, pre-admission, emergency-department, or other arrest occurring before ICU admission; an arrest during the first 24 h; or an event after the prespecified outcome window. |

**5.4 IHCA event record and final classification**

| **Final IHCA status** | [ ] Yes [ ] No [ ] Unclear before adjudication |
| --- | --- |
| **IHCA date/time** | ____ / ____ / ______ ____:____ |
| **Time from ICU admission** | ________ h |
| **Hospital location** | ____________________________________________ |
| **Exact qualifying term or short phrase** | ________________________________________________________________________________ |
| **Source type and locator** | ________________________________________________________________________________ |
| **First documented rhythm, if available** | [ ] Asystole [ ] PEA [ ] VF [ ] pVT [ ] Other [ ] Unknown |
| **Resuscitation attempted** | [ ] Yes [ ] No [ ] Not documented |
| **Return of spontaneous circulation** | [ ] Yes [ ] No [ ] Not applicable or unknown |
| **Temporal eligibility** | [ ] After 24 h and within outcome window [ ] Outside window [ ] Unclear |
| **Notes** | ________________________________________________________________________________ ________________________________________________________________________________ |

**6. Verification and adjudication**

The initial extractor and verifier should complete their assessments before discrepancies are discussed. Occasional discrepancies in predictor abstraction or IHCA ascertainment are adjudicated by Yuqian Li following re-evaluation of the original source documentation, and only the verified or adjudicated determination is transferred to the analysis dataset.

| **Field** | **Entry** | **Field** | **Entry** |
| --- | --- | --- | --- |
| Initial extractor | ____________________________ | Date | ____ / ____ / ______ |
| Verifier | ____________________________ | Date | ____ / ____ / ______ |
| Predictor discrepancies identified | [ ] No [ ] Yes | IHCA disagreement identified | [ ] No [ ] Yes |
| Variables or outcome requiring review | ________________________________________________________________________________ | Source records re-examined | [ ] Yes [ ] No |
| Adjudicator | Yuqian Li | Adjudication date | ____ / ____ / ______ |
| Final adjudicated determination | ________________________________________________________________________________ | Transferred to analysis dataset | [ ] Yes [ ] No |

**References**

1. Nolan JP, Berg RA, Andersen LW, et al. Cardiac arrest and cardiopulmonary resuscitation outcome reports: update of the Utstein resuscitation registry template for in-hospital cardiac arrest. Circulation. 2019;140:e746–e757. doi:10.1161/CIR.0000000000000710.

2. American Heart Association. What is cardiac arrest? https://www.heart.org/en/health-topics/cardiac-arrest/about-cardiac-arrest (accessed Aug 3, 2026).
