# Supplementary material for: Machine learning–driven prediction of in-hospital cardiac arrest among critically ill patients with traumatic spinal cord injury: a multicenter cohort study
Source: Front Cardiovasc Med. 2026 Sep 14;13:1818433. doi: 10.3389/fcvm.2026.1818433 (PMC13617107; doi:10.3389/fcvm.2026.1818433)
Supplement: Supplementary file 3 [file Table1.docx]

Supplementary Table 1 Prespecified ICD-9-CM and ICD-10-CM Diagnosis Codes for Identifying Traumatic Spinal Cord Injury

| Coding system | Code | Database code (no dot) | Code family | Official description | Anatomical region |
| --- | --- | --- | --- | --- | --- |
| ICD-9-CM | 806.00 | 80600 | 806.x | Closed fracture of C1-C4 level with unspecified spinal cord injury | Cervical |
| ICD-9-CM | 806.01 | 80601 | 806.x | Closed fracture of C1-C4 level with complete lesion of cord | Cervical |
| ICD-9-CM | 806.02 | 80602 | 806.x | Closed fracture of C1-C4 level with anterior cord syndrome | Cervical |
| ICD-9-CM | 806.03 | 80603 | 806.x | Closed fracture of C1-C4 level with central cord syndrome | Cervical |
| ICD-9-CM | 806.04 | 80604 | 806.x | Closed fracture of C1-C4 level with other specified spinal cord injury | Cervical |
| ICD-9-CM | 806.05 | 80605 | 806.x | Closed fracture of C5-C7 level with unspecified spinal cord injury | Cervical |
| ICD-9-CM | 806.06 | 80606 | 806.x | Closed fracture of C5-C7 level with complete lesion of cord | Cervical |
| ICD-9-CM | 806.07 | 80607 | 806.x | Closed fracture of C5-C7 level with anterior cord syndrome | Cervical |
| ICD-9-CM | 806.08 | 80608 | 806.x | Closed fracture of C5-C7 level with central cord syndrome | Cervical |
| ICD-9-CM | 806.09 | 80609 | 806.x | Closed fracture of C5-C7 level with other specified spinal cord injury | Cervical |
| ICD-9-CM | 806.10 | 80610 | 806.x | Open fracture of C1-C4 level with unspecified spinal cord injury | Cervical |
| ICD-9-CM | 806.11 | 80611 | 806.x | Open fracture of C1-C4 level with complete lesion of cord | Cervical |
| ICD-9-CM | 806.12 | 80612 | 806.x | Open fracture of C1-C4 level with anterior cord syndrome | Cervical |
| ICD-9-CM | 806.13 | 80613 | 806.x | Open fracture of C1-C4 level with central cord syndrome | Cervical |
| ICD-9-CM | 806.14 | 80614 | 806.x | Open fracture of C1-C4 level with other specified spinal cord injury | Cervical |
| ICD-9-CM | 806.15 | 80615 | 806.x | Open fracture of C5-C7 level with unspecified spinal cord injury | Cervical |
| ICD-9-CM | 806.16 | 80616 | 806.x | Open fracture of C5-C7 level with complete lesion of cord | Cervical |
| ICD-9-CM | 806.17 | 80617 | 806.x | Open fracture of C5-C7 level with anterior cord syndrome | Cervical |
| ICD-9-CM | 806.18 | 80618 | 806.x | Open fracture of C5-C7 level with central cord syndrome | Cervical |
| ICD-9-CM | 806.19 | 80619 | 806.x | Open fracture of C5-C7 level with other specified spinal cord injury | Cervical |
| ICD-9-CM | 806.20 | 80620 | 806.x | Closed fracture of T1-T6 level with unspecified spinal cord injury | Thoracic |
| ICD-9-CM | 806.21 | 80621 | 806.x | Closed fracture of T1-T6 level with complete lesion of cord | Thoracic |
| ICD-9-CM | 806.22 | 80622 | 806.x | Closed fracture of T1-T6 level with anterior cord syndrome | Thoracic |
| ICD-9-CM | 806.23 | 80623 | 806.x | Closed fracture of T1-T6 level with central cord syndrome | Thoracic |
| ICD-9-CM | 806.24 | 80624 | 806.x | Closed fracture of T1-T6 level with other specified spinal cord injury | Thoracic |
| ICD-9-CM | 806.25 | 80625 | 806.x | Closed fracture of T7-T12 level with unspecified spinal cord injury | Thoracic |
| ICD-9-CM | 806.26 | 80626 | 806.x | Closed fracture of T7-T12 level with complete lesion of cord | Thoracic |
| ICD-9-CM | 806.27 | 80627 | 806.x | Closed fracture of T7-T12 level with anterior cord syndrome | Thoracic |
| ICD-9-CM | 806.28 | 80628 | 806.x | Closed fracture of T7-T12 level with central cord syndrome | Thoracic |
| ICD-9-CM | 806.29 | 80629 | 806.x | Closed fracture of T7-T12 level with other specified spinal cord injury | Thoracic |
| ICD-9-CM | 806.30 | 80630 | 806.x | Open fracture of T1-T6 level with unspecified spinal cord injury | Thoracic |
| ICD-9-CM | 806.31 | 80631 | 806.x | Open fracture of T1-T6 level with complete lesion of cord | Thoracic |
| ICD-9-CM | 806.32 | 80632 | 806.x | Open fracture of T1-T6 level with anterior cord syndrome | Thoracic |
| ICD-9-CM | 806.33 | 80633 | 806.x | Open fracture of T1-T6 level with central cord syndrome | Thoracic |
| ICD-9-CM | 806.34 | 80634 | 806.x | Open fracture of T1-T6 level with other specified spinal cord injury | Thoracic |
| ICD-9-CM | 806.35 | 80635 | 806.x | Open fracture of T7-T12 level with unspecified spinal cord injury | Thoracic |
| ICD-9-CM | 806.36 | 80636 | 806.x | Open fracture of T7-T12 level with complete lesion of cord | Thoracic |
| ICD-9-CM | 806.37 | 80637 | 806.x | Open fracture of T7-T12 level with anterior cord syndrome | Thoracic |
| ICD-9-CM | 806.38 | 80638 | 806.x | Open fracture of T7-T12 level with central cord syndrome | Thoracic |
| ICD-9-CM | 806.39 | 80639 | 806.x | Open fracture of T7-T12 level with other specified spinal cord injury | Thoracic |
| ICD-9-CM | 806.4 | 8064 | 806.x | Closed fracture of lumbar spine with spinal cord injury | Lumbar |
| ICD-9-CM | 806.5 | 8065 | 806.x | Open fracture of lumbar spine with spinal cord injury | Lumbar |
| ICD-9-CM | 806.60 | 80660 | 806.x | Closed fracture of sacrum and coccyx with unspecified spinal cord injury | Sacral/coccygeal |
| ICD-9-CM | 806.61 | 80661 | 806.x | Closed fracture of sacrum and coccyx with complete cauda equina lesion | Sacral/coccygeal |
| ICD-9-CM | 806.62 | 80662 | 806.x | Closed fracture of sacrum and coccyx with other cauda equina injury | Sacral/coccygeal |
| ICD-9-CM | 806.69 | 80669 | 806.x | Closed fracture of sacrum and coccyx with other spinal cord injury | Sacral/coccygeal |
| ICD-9-CM | 806.70 | 80670 | 806.x | Open fracture of sacrum and coccyx with unspecified spinal cord injury | Sacral/coccygeal |
| ICD-9-CM | 806.71 | 80671 | 806.x | Open fracture of sacrum and coccyx with complete cauda equina lesion | Sacral/coccygeal |
| ICD-9-CM | 806.72 | 80672 | 806.x | Open fracture of sacrum and coccyx with other cauda equina injury | Sacral/coccygeal |
| ICD-9-CM | 806.79 | 80679 | 806.x | Open fracture of sacrum and coccyx with other spinal cord injury | Sacral/coccygeal |
| ICD-9-CM | 806.8 | 8068 | 806.x | Closed fracture of unspecified vertebral column with spinal cord injury | Unspecified |
| ICD-9-CM | 806.9 | 8069 | 806.x | Open fracture of unspecified vertebral column with spinal cord injury | Unspecified |
| ICD-9-CM | 952.00 | 95200 | 952.x | C1-C4 level with unspecified spinal cord injury | Cervical |
| ICD-9-CM | 952.01 | 95201 | 952.x | C1-C4 level with complete lesion of spinal cord | Cervical |
| ICD-9-CM | 952.02 | 95202 | 952.x | C1-C4 level with anterior cord syndrome | Cervical |
| ICD-9-CM | 952.03 | 95203 | 952.x | C1-C4 level with central cord syndrome | Cervical |
| ICD-9-CM | 952.04 | 95204 | 952.x | C1-C4 level with other specified spinal cord injury | Cervical |
| ICD-9-CM | 952.05 | 95205 | 952.x | C5-C7 level with unspecified spinal cord injury | Cervical |
| ICD-9-CM | 952.06 | 95206 | 952.x | C5-C7 level with complete lesion of spinal cord | Cervical |
| ICD-9-CM | 952.07 | 95207 | 952.x | C5-C7 level with anterior cord syndrome | Cervical |
| ICD-9-CM | 952.08 | 95208 | 952.x | C5-C7 level with central cord syndrome | Cervical |
| ICD-9-CM | 952.09 | 95209 | 952.x | C5-C7 level with other specified spinal cord injury | Cervical |
| ICD-9-CM | 952.10 | 95210 | 952.x | T1-T6 level with unspecified spinal cord injury | Thoracic |
| ICD-9-CM | 952.11 | 95211 | 952.x | T1-T6 level with complete lesion of spinal cord | Thoracic |
| ICD-9-CM | 952.12 | 95212 | 952.x | T1-T6 level with anterior cord syndrome | Thoracic |
| ICD-9-CM | 952.13 | 95213 | 952.x | T1-T6 level with central cord syndrome | Thoracic |
| ICD-9-CM | 952.14 | 95214 | 952.x | T1-T6 level with other specified spinal cord injury | Thoracic |
| ICD-9-CM | 952.15 | 95215 | 952.x | T7-T12 level with unspecified spinal cord injury | Thoracic |
| ICD-9-CM | 952.16 | 95216 | 952.x | T7-T12 level with complete lesion of spinal cord | Thoracic |
| ICD-9-CM | 952.17 | 95217 | 952.x | T7-T12 level with anterior cord syndrome | Thoracic |
| ICD-9-CM | 952.18 | 95218 | 952.x | T7-T12 level with central cord syndrome | Thoracic |
| ICD-9-CM | 952.19 | 95219 | 952.x | T7-T12 level with other specified spinal cord injury | Thoracic |
| ICD-9-CM | 952.2 | 9522 | 952.x | Lumbar spinal cord injury without evidence of spinal bone injury | Lumbar |
| ICD-9-CM | 952.3 | 9523 | 952.x | Sacral spinal cord injury without evidence of spinal bone injury | Sacral/coccygeal |
| ICD-9-CM | 952.4 | 9524 | 952.x | Cauda equina spinal cord injury without evidence of spinal bone injury | Cauda equina |
| ICD-9-CM | 952.8 | 9528 | 952.x | Multiple sites of spinal cord injury without evidence of spinal bone injury | Multiple levels |
| ICD-9-CM | 952.9 | 9529 | 952.x | Unspecified site of spinal cord injury without evidence of spinal bone injury | Unspecified |
| ICD-10-CM | S14.0XXA | S140XXA | S14.0* | Concussion and edema of cervical spinal cord, initial encounter | Cervical |
| ICD-10-CM | S14.0XXD | S140XXD | S14.0* | Concussion and edema of cervical spinal cord, subsequent encounter | Cervical |
| ICD-10-CM | S14.0XXS | S140XXS | S14.0* | Concussion and edema of cervical spinal cord, sequela | Cervical |
| ICD-10-CM | S14.101A | S14101A | S14.1* | Unspecified injury at C1 level of cervical spinal cord, initial encounter | Cervical |
| ICD-10-CM | S14.101D | S14101D | S14.1* | Unspecified injury at C1 level of cervical spinal cord, subsequent encounter | Cervical |
| ICD-10-CM | S14.101S | S14101S | S14.1* | Unspecified injury at C1 level of cervical spinal cord, sequela | Cervical |
| ICD-10-CM | S14.102A | S14102A | S14.1* | Unspecified injury at C2 level of cervical spinal cord, initial encounter | Cervical |
| ICD-10-CM | S14.102D | S14102D | S14.1* | Unspecified injury at C2 level of cervical spinal cord, subsequent encounter | Cervical |
| ICD-10-CM | S14.102S | S14102S | S14.1* | Unspecified injury at C2 level of cervical spinal cord, sequela | Cervical |
| ICD-10-CM | S14.103A | S14103A | S14.1* | Unspecified injury at C3 level of cervical spinal cord, initial encounter | Cervical |
| ICD-10-CM | S14.103D | S14103D | S14.1* | Unspecified injury at C3 level of cervical spinal cord, subsequent encounter | Cervical |
| ICD-10-CM | S14.103S | S14103S | S14.1* | Unspecified injury at C3 level of cervical spinal cord, sequela | Cervical |
| ICD-10-CM | S14.104A | S14104A | S14.1* | Unspecified injury at C4 level of cervical spinal cord, initial encounter | Cervical |
| ICD-10-CM | S14.104D | S14104D | S14.1* | Unspecified injury at C4 level of cervical spinal cord, subsequent encounter | Cervical |
| ICD-10-CM | S14.104S | S14104S | S14.1* | Unspecified injury at C4 level of cervical spinal cord, sequela | Cervical |
| ICD-10-CM | S14.105A | S14105A | S14.1* | Unspecified injury at C5 level of cervical spinal cord, initial encounter | Cervical |
| ICD-10-CM | S14.105D | S14105D | S14.1* | Unspecified injury at C5 level of cervical spinal cord, subsequent encounter | Cervical |
| ICD-10-CM | S14.105S | S14105S | S14.1* | Unspecified injury at C5 level of cervical spinal cord, sequela | Cervical |
| ICD-10-CM | S14.106A | S14106A | S14.1* | Unspecified injury at C6 level of cervical spinal cord, initial encounter | Cervical |
| ICD-10-CM | S14.106D | S14106D | S14.1* | Unspecified injury at C6 level of cervical spinal cord, subsequent encounter | Cervical |
| ICD-10-CM | S14.106S | S14106S | S14.1* | Unspecified injury at C6 level of cervical spinal cord, sequela | Cervical |
| ICD-10-CM | S14.107A | S14107A | S14.1* | Unspecified injury at C7 level of cervical spinal cord, initial encounter | Cervical |
| ICD-10-CM | S14.107D | S14107D | S14.1* | Unspecified injury at C7 level of cervical spinal cord, subsequent encounter | Cervical |
| ICD-10-CM | S14.107S | S14107S | S14.1* | Unspecified injury at C7 level of cervical spinal cord, sequela | Cervical |
| ICD-10-CM | S14.108A | S14108A | S14.1* | Unspecified injury at C8 level of cervical spinal cord, initial encounter | Cervical |
| ICD-10-CM | S14.108D | S14108D | S14.1* | Unspecified injury at C8 level of cervical spinal cord, subsequent encounter | Cervical |
| ICD-10-CM | S14.108S | S14108S | S14.1* | Unspecified injury at C8 level of cervical spinal cord, sequela | Cervical |
| ICD-10-CM | S14.109A | S14109A | S14.1* | Unspecified injury at unspecified level of cervical spinal cord, initial encounter | Cervical |
| ICD-10-CM | S14.109D | S14109D | S14.1* | Unspecified injury at unspecified level of cervical spinal cord, subsequent encounter | Cervical |
| ICD-10-CM | S14.109S | S14109S | S14.1* | Unspecified injury at unspecified level of cervical spinal cord, sequela | Cervical |
| ICD-10-CM | S14.111A | S14111A | S14.1* | Complete lesion at C1 level of cervical spinal cord, initial encounter | Cervical |
| ICD-10-CM | S14.111D | S14111D | S14.1* | Complete lesion at C1 level of cervical spinal cord, subsequent encounter | Cervical |
| ICD-10-CM | S14.111S | S14111S | S14.1* | Complete lesion at C1 level of cervical spinal cord, sequela | Cervical |
| ICD-10-CM | S14.112A | S14112A | S14.1* | Complete lesion at C2 level of cervical spinal cord, initial encounter | Cervical |
| ICD-10-CM | S14.112D | S14112D | S14.1* | Complete lesion at C2 level of cervical spinal cord, subsequent encounter | Cervical |
| ICD-10-CM | S14.112S | S14112S | S14.1* | Complete lesion at C2 level of cervical spinal cord, sequela | Cervical |
| ICD-10-CM | S14.113A | S14113A | S14.1* | Complete lesion at C3 level of cervical spinal cord, initial encounter | Cervical |
| ICD-10-CM | S14.113D | S14113D | S14.1* | Complete lesion at C3 level of cervical spinal cord, subsequent encounter | Cervical |
| ICD-10-CM | S14.113S | S14113S | S14.1* | Complete lesion at C3 level of cervical spinal cord, sequela | Cervical |
| ICD-10-CM | S14.114A | S14114A | S14.1* | Complete lesion at C4 level of cervical spinal cord, initial encounter | Cervical |
| ICD-10-CM | S14.114D | S14114D | S14.1* | Complete lesion at C4 level of cervical spinal cord, subsequent encounter | Cervical |
| ICD-10-CM | S14.114S | S14114S | S14.1* | Complete lesion at C4 level of cervical spinal cord, sequela | Cervical |
| ICD-10-CM | S14.115A | S14115A | S14.1* | Complete lesion at C5 level of cervical spinal cord, initial encounter | Cervical |
| ICD-10-CM | S14.115D | S14115D | S14.1* | Complete lesion at C5 level of cervical spinal cord, subsequent encounter | Cervical |
| ICD-10-CM | S14.115S | S14115S | S14.1* | Complete lesion at C5 level of cervical spinal cord, sequela | Cervical |
| ICD-10-CM | S14.116A | S14116A | S14.1* | Complete lesion at C6 level of cervical spinal cord, initial encounter | Cervical |
| ICD-10-CM | S14.116D | S14116D | S14.1* | Complete lesion at C6 level of cervical spinal cord, subsequent encounter | Cervical |
| ICD-10-CM | S14.116S | S14116S | S14.1* | Complete lesion at C6 level of cervical spinal cord, sequela | Cervical |
| ICD-10-CM | S14.117A | S14117A | S14.1* | Complete lesion at C7 level of cervical spinal cord, initial encounter | Cervical |
| ICD-10-CM | S14.117D | S14117D | S14.1* | Complete lesion at C7 level of cervical spinal cord, subsequent encounter | Cervical |
| ICD-10-CM | S14.117S | S14117S | S14.1* | Complete lesion at C7 level of cervical spinal cord, sequela | Cervical |
| ICD-10-CM | S14.118A | S14118A | S14.1* | Complete lesion at C8 level of cervical spinal cord, initial encounter | Cervical |
| ICD-10-CM | S14.118D | S14118D | S14.1* | Complete lesion at C8 level of cervical spinal cord, subsequent encounter | Cervical |
| ICD-10-CM | S14.118S | S14118S | S14.1* | Complete lesion at C8 level of cervical spinal cord, sequela | Cervical |
| ICD-10-CM | S14.119A | S14119A | S14.1* | Complete lesion at unspecified level of cervical spinal cord, initial encounter | Cervical |
| ICD-10-CM | S14.119D | S14119D | S14.1* | Complete lesion at unspecified level of cervical spinal cord, subsequent encounter | Cervical |
| ICD-10-CM | S14.119S | S14119S | S14.1* | Complete lesion at unspecified level of cervical spinal cord, sequela | Cervical |
| ICD-10-CM | S14.121A | S14121A | S14.1* | Central cord syndrome at C1 level of cervical spinal cord, initial encounter | Cervical |
| ICD-10-CM | S14.121D | S14121D | S14.1* | Central cord syndrome at C1 level of cervical spinal cord, subsequent encounter | Cervical |
| ICD-10-CM | S14.121S | S14121S | S14.1* | Central cord syndrome at C1 level of cervical spinal cord, sequela | Cervical |
| ICD-10-CM | S14.122A | S14122A | S14.1* | Central cord syndrome at C2 level of cervical spinal cord, initial encounter | Cervical |
| ICD-10-CM | S14.122D | S14122D | S14.1* | Central cord syndrome at C2 level of cervical spinal cord, subsequent encounter | Cervical |
| ICD-10-CM | S14.122S | S14122S | S14.1* | Central cord syndrome at C2 level of cervical spinal cord, sequela | Cervical |
| ICD-10-CM | S14.123A | S14123A | S14.1* | Central cord syndrome at C3 level of cervical spinal cord, initial encounter | Cervical |
| ICD-10-CM | S14.123D | S14123D | S14.1* | Central cord syndrome at C3 level of cervical spinal cord, subsequent encounter | Cervical |
| ICD-10-CM | S14.123S | S14123S | S14.1* | Central cord syndrome at C3 level of cervical spinal cord, sequela | Cervical |
| ICD-10-CM | S14.124A | S14124A | S14.1* | Central cord syndrome at C4 level of cervical spinal cord, initial encounter | Cervical |
| ICD-10-CM | S14.124D | S14124D | S14.1* | Central cord syndrome at C4 level of cervical spinal cord, subsequent encounter | Cervical |
| ICD-10-CM | S14.124S | S14124S | S14.1* | Central cord syndrome at C4 level of cervical spinal cord, sequela | Cervical |
| ICD-10-CM | S14.125A | S14125A | S14.1* | Central cord syndrome at C5 level of cervical spinal cord, initial encounter | Cervical |
| ICD-10-CM | S14.125D | S14125D | S14.1* | Central cord syndrome at C5 level of cervical spinal cord, subsequent encounter | Cervical |
| ICD-10-CM | S14.125S | S14125S | S14.1* | Central cord syndrome at C5 level of cervical spinal cord, sequela | Cervical |
| ICD-10-CM | S14.126A | S14126A | S14.1* | Central cord syndrome at C6 level of cervical spinal cord, initial encounter | Cervical |
| ICD-10-CM | S14.126D | S14126D | S14.1* | Central cord syndrome at C6 level of cervical spinal cord, subsequent encounter | Cervical |
| ICD-10-CM | S14.126S | S14126S | S14.1* | Central cord syndrome at C6 level of cervical spinal cord, sequela | Cervical |
| ICD-10-CM | S14.127A | S14127A | S14.1* | Central cord syndrome at C7 level of cervical spinal cord, initial encounter | Cervical |
| ICD-10-CM | S14.127D | S14127D | S14.1* | Central cord syndrome at C7 level of cervical spinal cord, subsequent encounter | Cervical |
| ICD-10-CM | S14.127S | S14127S | S14.1* | Central cord syndrome at C7 level of cervical spinal cord, sequela | Cervical |
| ICD-10-CM | S14.128A | S14128A | S14.1* | Central cord syndrome at C8 level of cervical spinal cord, initial encounter | Cervical |
| ICD-10-CM | S14.128D | S14128D | S14.1* | Central cord syndrome at C8 level of cervical spinal cord, subsequent encounter | Cervical |
| ICD-10-CM | S14.128S | S14128S | S14.1* | Central cord syndrome at C8 level of cervical spinal cord, sequela | Cervical |
| ICD-10-CM | S14.129A | S14129A | S14.1* | Central cord syndrome at unspecified level of cervical spinal cord, initial encounter | Cervical |
| ICD-10-CM | S14.129D | S14129D | S14.1* | Central cord syndrome at unspecified level of cervical spinal cord, subsequent encounter | Cervical |
| ICD-10-CM | S14.129S | S14129S | S14.1* | Central cord syndrome at unspecified level of cervical spinal cord, sequela | Cervical |
| ICD-10-CM | S14.131A | S14131A | S14.1* | Anterior cord syndrome at C1 level of cervical spinal cord, initial encounter | Cervical |
| ICD-10-CM | S14.131D | S14131D | S14.1* | Anterior cord syndrome at C1 level of cervical spinal cord, subsequent encounter | Cervical |
| ICD-10-CM | S14.131S | S14131S | S14.1* | Anterior cord syndrome at C1 level of cervical spinal cord, sequela | Cervical |
| ICD-10-CM | S14.132A | S14132A | S14.1* | Anterior cord syndrome at C2 level of cervical spinal cord, initial encounter | Cervical |
| ICD-10-CM | S14.132D | S14132D | S14.1* | Anterior cord syndrome at C2 level of cervical spinal cord, subsequent encounter | Cervical |
| ICD-10-CM | S14.132S | S14132S | S14.1* | Anterior cord syndrome at C2 level of cervical spinal cord, sequela | Cervical |
| ICD-10-CM | S14.133A | S14133A | S14.1* | Anterior cord syndrome at C3 level of cervical spinal cord, initial encounter | Cervical |
| ICD-10-CM | S14.133D | S14133D | S14.1* | Anterior cord syndrome at C3 level of cervical spinal cord, subsequent encounter | Cervical |
| ICD-10-CM | S14.133S | S14133S | S14.1* | Anterior cord syndrome at C3 level of cervical spinal cord, sequela | Cervical |
| ICD-10-CM | S14.134A | S14134A | S14.1* | Anterior cord syndrome at C4 level of cervical spinal cord, initial encounter | Cervical |
| ICD-10-CM | S14.134D | S14134D | S14.1* | Anterior cord syndrome at C4 level of cervical spinal cord, subsequent encounter | Cervical |
| ICD-10-CM | S14.134S | S14134S | S14.1* | Anterior cord syndrome at C4 level of cervical spinal cord, sequela | Cervical |
| ICD-10-CM | S14.135A | S14135A | S14.1* | Anterior cord syndrome at C5 level of cervical spinal cord, initial encounter | Cervical |
| ICD-10-CM | S14.135D | S14135D | S14.1* | Anterior cord syndrome at C5 level of cervical spinal cord, subsequent encounter | Cervical |
| ICD-10-CM | S14.135S | S14135S | S14.1* | Anterior cord syndrome at C5 level of cervical spinal cord, sequela | Cervical |
| ICD-10-CM | S14.136A | S14136A | S14.1* | Anterior cord syndrome at C6 level of cervical spinal cord, initial encounter | Cervical |
| ICD-10-CM | S14.136D | S14136D | S14.1* | Anterior cord syndrome at C6 level of cervical spinal cord, subsequent encounter | Cervical |
| ICD-10-CM | S14.136S | S14136S | S14.1* | Anterior cord syndrome at C6 level of cervical spinal cord, sequela | Cervical |
| ICD-10-CM | S14.137A | S14137A | S14.1* | Anterior cord syndrome at C7 level of cervical spinal cord, initial encounter | Cervical |
| ICD-10-CM | S14.137D | S14137D | S14.1* | Anterior cord syndrome at C7 level of cervical spinal cord, subsequent encounter | Cervical |
| ICD-10-CM | S14.137S | S14137S | S14.1* | Anterior cord syndrome at C7 level of cervical spinal cord, sequela | Cervical |
| ICD-10-CM | S14.138A | S14138A | S14.1* | Anterior cord syndrome at C8 level of cervical spinal cord, initial encounter | Cervical |
| ICD-10-CM | S14.138D | S14138D | S14.1* | Anterior cord syndrome at C8 level of cervical spinal cord, subsequent encounter | Cervical |
| ICD-10-CM | S14.138S | S14138S | S14.1* | Anterior cord syndrome at C8 level of cervical spinal cord, sequela | Cervical |
| ICD-10-CM | S14.139A | S14139A | S14.1* | Anterior cord syndrome at unspecified level of cervical spinal cord, initial encounter | Cervical |
| ICD-10-CM | S14.139D | S14139D | S14.1* | Anterior cord syndrome at unspecified level of cervical spinal cord, subsequent encounter | Cervical |
| ICD-10-CM | S14.139S | S14139S | S14.1* | Anterior cord syndrome at unspecified level of cervical spinal cord, sequela | Cervical |
| ICD-10-CM | S14.141A | S14141A | S14.1* | Brown-Sequard syndrome at C1 level of cervical spinal cord, initial encounter | Cervical |
| ICD-10-CM | S14.141D | S14141D | S14.1* | Brown-Sequard syndrome at C1 level of cervical spinal cord, subsequent encounter | Cervical |
| ICD-10-CM | S14.141S | S14141S | S14.1* | Brown-Sequard syndrome at C1 level of cervical spinal cord, sequela | Cervical |
| ICD-10-CM | S14.142A | S14142A | S14.1* | Brown-Sequard syndrome at C2 level of cervical spinal cord, initial encounter | Cervical |
| ICD-10-CM | S14.142D | S14142D | S14.1* | Brown-Sequard syndrome at C2 level of cervical spinal cord, subsequent encounter | Cervical |
| ICD-10-CM | S14.142S | S14142S | S14.1* | Brown-Sequard syndrome at C2 level of cervical spinal cord, sequela | Cervical |
| ICD-10-CM | S14.143A | S14143A | S14.1* | Brown-Sequard syndrome at C3 level of cervical spinal cord, initial encounter | Cervical |
| ICD-10-CM | S14.143D | S14143D | S14.1* | Brown-Sequard syndrome at C3 level of cervical spinal cord, subsequent encounter | Cervical |
| ICD-10-CM | S14.143S | S14143S | S14.1* | Brown-Sequard syndrome at C3 level of cervical spinal cord, sequela | Cervical |
| ICD-10-CM | S14.144A | S14144A | S14.1* | Brown-Sequard syndrome at C4 level of cervical spinal cord, initial encounter | Cervical |
| ICD-10-CM | S14.144D | S14144D | S14.1* | Brown-Sequard syndrome at C4 level of cervical spinal cord, subsequent encounter | Cervical |
| ICD-10-CM | S14.144S | S14144S | S14.1* | Brown-Sequard syndrome at C4 level of cervical spinal cord, sequela | Cervical |
| ICD-10-CM | S14.145A | S14145A | S14.1* | Brown-Sequard syndrome at C5 level of cervical spinal cord, initial encounter | Cervical |
| ICD-10-CM | S14.145D | S14145D | S14.1* | Brown-Sequard syndrome at C5 level of cervical spinal cord, subsequent encounter | Cervical |
| ICD-10-CM | S14.145S | S14145S | S14.1* | Brown-Sequard syndrome at C5 level of cervical spinal cord, sequela | Cervical |
| ICD-10-CM | S14.146A | S14146A | S14.1* | Brown-Sequard syndrome at C6 level of cervical spinal cord, initial encounter | Cervical |
| ICD-10-CM | S14.146D | S14146D | S14.1* | Brown-Sequard syndrome at C6 level of cervical spinal cord, subsequent encounter | Cervical |
| ICD-10-CM | S14.146S | S14146S | S14.1* | Brown-Sequard syndrome at C6 level of cervical spinal cord, sequela | Cervical |
| ICD-10-CM | S14.147A | S14147A | S14.1* | Brown-Sequard syndrome at C7 level of cervical spinal cord, initial encounter | Cervical |
| ICD-10-CM | S14.147D | S14147D | S14.1* | Brown-Sequard syndrome at C7 level of cervical spinal cord, subsequent encounter | Cervical |
| ICD-10-CM | S14.147S | S14147S | S14.1* | Brown-Sequard syndrome at C7 level of cervical spinal cord, sequela | Cervical |
| ICD-10-CM | S14.148A | S14148A | S14.1* | Brown-Sequard syndrome at C8 level of cervical spinal cord, initial encounter | Cervical |
| ICD-10-CM | S14.148D | S14148D | S14.1* | Brown-Sequard syndrome at C8 level of cervical spinal cord, subsequent encounter | Cervical |
| ICD-10-CM | S14.148S | S14148S | S14.1* | Brown-Sequard syndrome at C8 level of cervical spinal cord, sequela | Cervical |
| ICD-10-CM | S14.149A | S14149A | S14.1* | Brown-Sequard syndrome at unspecified level of cervical spinal cord, initial encounter | Cervical |
| ICD-10-CM | S14.149D | S14149D | S14.1* | Brown-Sequard syndrome at unspecified level of cervical spinal cord, subsequent encounter | Cervical |
| ICD-10-CM | S14.149S | S14149S | S14.1* | Brown-Sequard syndrome at unspecified level of cervical spinal cord, sequela | Cervical |
| ICD-10-CM | S14.151A | S14151A | S14.1* | Other incomplete lesion at C1 level of cervical spinal cord, initial encounter | Cervical |
| ICD-10-CM | S14.151D | S14151D | S14.1* | Other incomplete lesion at C1 level of cervical spinal cord, subsequent encounter | Cervical |
| ICD-10-CM | S14.151S | S14151S | S14.1* | Other incomplete lesion at C1 level of cervical spinal cord, sequela | Cervical |
| ICD-10-CM | S14.152A | S14152A | S14.1* | Other incomplete lesion at C2 level of cervical spinal cord, initial encounter | Cervical |
| ICD-10-CM | S14.152D | S14152D | S14.1* | Other incomplete lesion at C2 level of cervical spinal cord, subsequent encounter | Cervical |
| ICD-10-CM | S14.152S | S14152S | S14.1* | Other incomplete lesion at C2 level of cervical spinal cord, sequela | Cervical |
| ICD-10-CM | S14.153A | S14153A | S14.1* | Other incomplete lesion at C3 level of cervical spinal cord, initial encounter | Cervical |
| ICD-10-CM | S14.153D | S14153D | S14.1* | Other incomplete lesion at C3 level of cervical spinal cord, subsequent encounter | Cervical |
| ICD-10-CM | S14.153S | S14153S | S14.1* | Other incomplete lesion at C3 level of cervical spinal cord, sequela | Cervical |
| ICD-10-CM | S14.154A | S14154A | S14.1* | Other incomplete lesion at C4 level of cervical spinal cord, initial encounter | Cervical |
| ICD-10-CM | S14.154D | S14154D | S14.1* | Other incomplete lesion at C4 level of cervical spinal cord, subsequent encounter | Cervical |
| ICD-10-CM | S14.154S | S14154S | S14.1* | Other incomplete lesion at C4 level of cervical spinal cord, sequela | Cervical |
| ICD-10-CM | S14.155A | S14155A | S14.1* | Other incomplete lesion at C5 level of cervical spinal cord, initial encounter | Cervical |
| ICD-10-CM | S14.155D | S14155D | S14.1* | Other incomplete lesion at C5 level of cervical spinal cord, subsequent encounter | Cervical |
| ICD-10-CM | S14.155S | S14155S | S14.1* | Other incomplete lesion at C5 level of cervical spinal cord, sequela | Cervical |
| ICD-10-CM | S14.156A | S14156A | S14.1* | Other incomplete lesion at C6 level of cervical spinal cord, initial encounter | Cervical |
| ICD-10-CM | S14.156D | S14156D | S14.1* | Other incomplete lesion at C6 level of cervical spinal cord, subsequent encounter | Cervical |
| ICD-10-CM | S14.156S | S14156S | S14.1* | Other incomplete lesion at C6 level of cervical spinal cord, sequela | Cervical |
| ICD-10-CM | S14.157A | S14157A | S14.1* | Other incomplete lesion at C7 level of cervical spinal cord, initial encounter | Cervical |
| ICD-10-CM | S14.157D | S14157D | S14.1* | Other incomplete lesion at C7 level of cervical spinal cord, subsequent encounter | Cervical |
| ICD-10-CM | S14.157S | S14157S | S14.1* | Other incomplete lesion at C7 level of cervical spinal cord, sequela | Cervical |
| ICD-10-CM | S14.158A | S14158A | S14.1* | Other incomplete lesion at C8 level of cervical spinal cord, initial encounter | Cervical |
| ICD-10-CM | S14.158D | S14158D | S14.1* | Other incomplete lesion at C8 level of cervical spinal cord, subsequent encounter | Cervical |
| ICD-10-CM | S14.158S | S14158S | S14.1* | Other incomplete lesion at C8 level of cervical spinal cord, sequela | Cervical |
| ICD-10-CM | S14.159A | S14159A | S14.1* | Other incomplete lesion at unspecified level of cervical spinal cord, initial encounter | Cervical |
| ICD-10-CM | S14.159D | S14159D | S14.1* | Other incomplete lesion at unspecified level of cervical spinal cord, subsequent encounter | Cervical |
| ICD-10-CM | S14.159S | S14159S | S14.1* | Other incomplete lesion at unspecified level of cervical spinal cord, sequela | Cervical |
| ICD-10-CM | S24.0XXA | S240XXA | S24.0* | Concussion and edema of thoracic spinal cord, initial encounter | Thoracic |
| ICD-10-CM | S24.0XXD | S240XXD | S24.0* | Concussion and edema of thoracic spinal cord, subsequent encounter | Thoracic |
| ICD-10-CM | S24.0XXS | S240XXS | S24.0* | Concussion and edema of thoracic spinal cord, sequela | Thoracic |
| ICD-10-CM | S24.101A | S24101A | S24.1* | Unspecified injury at T1 level of thoracic spinal cord, initial encounter | Thoracic |
| ICD-10-CM | S24.101D | S24101D | S24.1* | Unspecified injury at T1 level of thoracic spinal cord, subsequent encounter | Thoracic |
| ICD-10-CM | S24.101S | S24101S | S24.1* | Unspecified injury at T1 level of thoracic spinal cord, sequela | Thoracic |
| ICD-10-CM | S24.102A | S24102A | S24.1* | Unspecified injury at T2-T6 level of thoracic spinal cord, initial encounter | Thoracic |
| ICD-10-CM | S24.102D | S24102D | S24.1* | Unspecified injury at T2-T6 level of thoracic spinal cord, subsequent encounter | Thoracic |
| ICD-10-CM | S24.102S | S24102S | S24.1* | Unspecified injury at T2-T6 level of thoracic spinal cord, sequela | Thoracic |
| ICD-10-CM | S24.103A | S24103A | S24.1* | Unspecified injury at T7-T10 level of thoracic spinal cord, initial encounter | Thoracic |
| ICD-10-CM | S24.103D | S24103D | S24.1* | Unspecified injury at T7-T10 level of thoracic spinal cord, subsequent encounter | Thoracic |
| ICD-10-CM | S24.103S | S24103S | S24.1* | Unspecified injury at T7-T10 level of thoracic spinal cord, sequela | Thoracic |
| ICD-10-CM | S24.104A | S24104A | S24.1* | Unspecified injury at T11-T12 level of thoracic spinal cord, initial encounter | Thoracic |
| ICD-10-CM | S24.104D | S24104D | S24.1* | Unspecified injury at T11-T12 level of thoracic spinal cord, subsequent encounter | Thoracic |
| ICD-10-CM | S24.104S | S24104S | S24.1* | Unspecified injury at T11-T12 level of thoracic spinal cord, sequela | Thoracic |
| ICD-10-CM | S24.109A | S24109A | S24.1* | Unspecified injury at unspecified level of thoracic spinal cord, initial encounter | Thoracic |
| ICD-10-CM | S24.109D | S24109D | S24.1* | Unspecified injury at unspecified level of thoracic spinal cord, subsequent encounter | Thoracic |
| ICD-10-CM | S24.109S | S24109S | S24.1* | Unspecified injury at unspecified level of thoracic spinal cord, sequela | Thoracic |
| ICD-10-CM | S24.111A | S24111A | S24.1* | Complete lesion at T1 level of thoracic spinal cord, initial encounter | Thoracic |
| ICD-10-CM | S24.111D | S24111D | S24.1* | Complete lesion at T1 level of thoracic spinal cord, subsequent encounter | Thoracic |
| ICD-10-CM | S24.111S | S24111S | S24.1* | Complete lesion at T1 level of thoracic spinal cord, sequela | Thoracic |
| ICD-10-CM | S24.112A | S24112A | S24.1* | Complete lesion at T2-T6 level of thoracic spinal cord, initial encounter | Thoracic |
| ICD-10-CM | S24.112D | S24112D | S24.1* | Complete lesion at T2-T6 level of thoracic spinal cord, subsequent encounter | Thoracic |
| ICD-10-CM | S24.112S | S24112S | S24.1* | Complete lesion at T2-T6 level of thoracic spinal cord, sequela | Thoracic |
| ICD-10-CM | S24.113A | S24113A | S24.1* | Complete lesion at T7-T10 level of thoracic spinal cord, initial encounter | Thoracic |
| ICD-10-CM | S24.113D | S24113D | S24.1* | Complete lesion at T7-T10 level of thoracic spinal cord, subsequent encounter | Thoracic |
| ICD-10-CM | S24.113S | S24113S | S24.1* | Complete lesion at T7-T10 level of thoracic spinal cord, sequela | Thoracic |
| ICD-10-CM | S24.114A | S24114A | S24.1* | Complete lesion at T11-T12 level of thoracic spinal cord, initial encounter | Thoracic |
| ICD-10-CM | S24.114D | S24114D | S24.1* | Complete lesion at T11-T12 level of thoracic spinal cord, subsequent encounter | Thoracic |
| ICD-10-CM | S24.114S | S24114S | S24.1* | Complete lesion at T11-T12 level of thoracic spinal cord, sequela | Thoracic |
| ICD-10-CM | S24.119A | S24119A | S24.1* | Complete lesion at unspecified level of thoracic spinal cord, initial encounter | Thoracic |
| ICD-10-CM | S24.119D | S24119D | S24.1* | Complete lesion at unspecified level of thoracic spinal cord, subsequent encounter | Thoracic |
| ICD-10-CM | S24.119S | S24119S | S24.1* | Complete lesion at unspecified level of thoracic spinal cord, sequela | Thoracic |
| ICD-10-CM | S24.131A | S24131A | S24.1* | Anterior cord syndrome at T1 level of thoracic spinal cord, initial encounter | Thoracic |
| ICD-10-CM | S24.131D | S24131D | S24.1* | Anterior cord syndrome at T1 level of thoracic spinal cord, subsequent encounter | Thoracic |
| ICD-10-CM | S24.131S | S24131S | S24.1* | Anterior cord syndrome at T1 level of thoracic spinal cord, sequela | Thoracic |
| ICD-10-CM | S24.132A | S24132A | S24.1* | Anterior cord syndrome at T2-T6 level of thoracic spinal cord, initial encounter | Thoracic |
| ICD-10-CM | S24.132D | S24132D | S24.1* | Anterior cord syndrome at T2-T6 level of thoracic spinal cord, subsequent encounter | Thoracic |
| ICD-10-CM | S24.132S | S24132S | S24.1* | Anterior cord syndrome at T2-T6 level of thoracic spinal cord, sequela | Thoracic |
| ICD-10-CM | S24.133A | S24133A | S24.1* | Anterior cord syndrome at T7-T10 level of thoracic spinal cord, initial encounter | Thoracic |
| ICD-10-CM | S24.133D | S24133D | S24.1* | Anterior cord syndrome at T7-T10 level of thoracic spinal cord, subsequent encounter | Thoracic |
| ICD-10-CM | S24.133S | S24133S | S24.1* | Anterior cord syndrome at T7-T10 level of thoracic spinal cord, sequela | Thoracic |
| ICD-10-CM | S24.134A | S24134A | S24.1* | Anterior cord syndrome at T11-T12 level of thoracic spinal cord, initial encounter | Thoracic |
| ICD-10-CM | S24.134D | S24134D | S24.1* | Anterior cord syndrome at T11-T12 level of thoracic spinal cord, subsequent encounter | Thoracic |
| ICD-10-CM | S24.134S | S24134S | S24.1* | Anterior cord syndrome at T11-T12 level of thoracic spinal cord, sequela | Thoracic |
| ICD-10-CM | S24.139A | S24139A | S24.1* | Anterior cord syndrome at unspecified level of thoracic spinal cord, initial encounter | Thoracic |
| ICD-10-CM | S24.139D | S24139D | S24.1* | Anterior cord syndrome at unspecified level of thoracic spinal cord, subsequent encounter | Thoracic |
| ICD-10-CM | S24.139S | S24139S | S24.1* | Anterior cord syndrome at unspecified level of thoracic spinal cord, sequela | Thoracic |
| ICD-10-CM | S24.141A | S24141A | S24.1* | Brown-Sequard syndrome at T1 level of thoracic spinal cord, initial encounter | Thoracic |
| ICD-10-CM | S24.141D | S24141D | S24.1* | Brown-Sequard syndrome at T1 level of thoracic spinal cord, subsequent encounter | Thoracic |
| ICD-10-CM | S24.141S | S24141S | S24.1* | Brown-Sequard syndrome at T1 level of thoracic spinal cord, sequela | Thoracic |
| ICD-10-CM | S24.142A | S24142A | S24.1* | Brown-Sequard syndrome at T2-T6 level of thoracic spinal cord, initial encounter | Thoracic |
| ICD-10-CM | S24.142D | S24142D | S24.1* | Brown-Sequard syndrome at T2-T6 level of thoracic spinal cord, subsequent encounter | Thoracic |
| ICD-10-CM | S24.142S | S24142S | S24.1* | Brown-Sequard syndrome at T2-T6 level of thoracic spinal cord, sequela | Thoracic |
| ICD-10-CM | S24.143A | S24143A | S24.1* | Brown-Sequard syndrome at T7-T10 level of thoracic spinal cord, initial encounter | Thoracic |
| ICD-10-CM | S24.143D | S24143D | S24.1* | Brown-Sequard syndrome at T7-T10 level of thoracic spinal cord, subsequent encounter | Thoracic |
| ICD-10-CM | S24.143S | S24143S | S24.1* | Brown-Sequard syndrome at T7-T10 level of thoracic spinal cord, sequela | Thoracic |
| ICD-10-CM | S24.144A | S24144A | S24.1* | Brown-Sequard syndrome at T11-T12 level of thoracic spinal cord, initial encounter | Thoracic |
| ICD-10-CM | S24.144D | S24144D | S24.1* | Brown-Sequard syndrome at T11-T12 level of thoracic spinal cord, subsequent encounter | Thoracic |
| ICD-10-CM | S24.144S | S24144S | S24.1* | Brown-Sequard syndrome at T11-T12 level of thoracic spinal cord, sequela | Thoracic |
| ICD-10-CM | S24.149A | S24149A | S24.1* | Brown-Sequard syndrome at unspecified level of thoracic spinal cord, initial encounter | Thoracic |
| ICD-10-CM | S24.149D | S24149D | S24.1* | Brown-Sequard syndrome at unspecified level of thoracic spinal cord, subsequent encounter | Thoracic |
| ICD-10-CM | S24.149S | S24149S | S24.1* | Brown-Sequard syndrome at unspecified level of thoracic spinal cord, sequela | Thoracic |
| ICD-10-CM | S24.151A | S24151A | S24.1* | Other incomplete lesion at T1 level of thoracic spinal cord, initial encounter | Thoracic |
| ICD-10-CM | S24.151D | S24151D | S24.1* | Other incomplete lesion at T1 level of thoracic spinal cord, subsequent encounter | Thoracic |
| ICD-10-CM | S24.151S | S24151S | S24.1* | Other incomplete lesion at T1 level of thoracic spinal cord, sequela | Thoracic |
| ICD-10-CM | S24.152A | S24152A | S24.1* | Other incomplete lesion at T2-T6 level of thoracic spinal cord, initial encounter | Thoracic |
| ICD-10-CM | S24.152D | S24152D | S24.1* | Other incomplete lesion at T2-T6 level of thoracic spinal cord, subsequent encounter | Thoracic |
| ICD-10-CM | S24.152S | S24152S | S24.1* | Other incomplete lesion at T2-T6 level of thoracic spinal cord, sequela | Thoracic |
| ICD-10-CM | S24.153A | S24153A | S24.1* | Other incomplete lesion at T7-T10 level of thoracic spinal cord, initial encounter | Thoracic |
| ICD-10-CM | S24.153D | S24153D | S24.1* | Other incomplete lesion at T7-T10 level of thoracic spinal cord, subsequent encounter | Thoracic |
| ICD-10-CM | S24.153S | S24153S | S24.1* | Other incomplete lesion at T7-T10 level of thoracic spinal cord, sequela | Thoracic |
| ICD-10-CM | S24.154A | S24154A | S24.1* | Other incomplete lesion at T11-T12 level of thoracic spinal cord, initial encounter | Thoracic |
| ICD-10-CM | S24.154D | S24154D | S24.1* | Other incomplete lesion at T11-T12 level of thoracic spinal cord, subsequent encounter | Thoracic |
| ICD-10-CM | S24.154S | S24154S | S24.1* | Other incomplete lesion at T11-T12 level of thoracic spinal cord, sequela | Thoracic |
| ICD-10-CM | S24.159A | S24159A | S24.1* | Other incomplete lesion at unspecified level of thoracic spinal cord, initial encounter | Thoracic |
| ICD-10-CM | S24.159D | S24159D | S24.1* | Other incomplete lesion at unspecified level of thoracic spinal cord, subsequent encounter | Thoracic |
| ICD-10-CM | S24.159S | S24159S | S24.1* | Other incomplete lesion at unspecified level of thoracic spinal cord, sequela | Thoracic |
| ICD-10-CM | S34.01XA | S3401XA | S34.0* | Concussion and edema of lumbar spinal cord, initial encounter | Lumbar |
| ICD-10-CM | S34.01XD | S3401XD | S34.0* | Concussion and edema of lumbar spinal cord, subsequent encounter | Lumbar |
| ICD-10-CM | S34.01XS | S3401XS | S34.0* | Concussion and edema of lumbar spinal cord, sequela | Lumbar |
| ICD-10-CM | S34.02XA | S3402XA | S34.0* | Concussion and edema of sacral spinal cord, initial encounter | Sacral/coccygeal |
| ICD-10-CM | S34.02XD | S3402XD | S34.0* | Concussion and edema of sacral spinal cord, subsequent encounter | Sacral/coccygeal |
| ICD-10-CM | S34.02XS | S3402XS | S34.0* | Concussion and edema of sacral spinal cord, sequela | Sacral/coccygeal |
| ICD-10-CM | S34.101A | S34101A | S34.1* | Unspecified injury to L1 level of lumbar spinal cord, initial encounter | Lumbar |
| ICD-10-CM | S34.101D | S34101D | S34.1* | Unspecified injury to L1 level of lumbar spinal cord, subsequent encounter | Lumbar |
| ICD-10-CM | S34.101S | S34101S | S34.1* | Unspecified injury to L1 level of lumbar spinal cord, sequela | Lumbar |
| ICD-10-CM | S34.102A | S34102A | S34.1* | Unspecified injury to L2 level of lumbar spinal cord, initial encounter | Lumbar |
| ICD-10-CM | S34.102D | S34102D | S34.1* | Unspecified injury to L2 level of lumbar spinal cord, subsequent encounter | Lumbar |
| ICD-10-CM | S34.102S | S34102S | S34.1* | Unspecified injury to L2 level of lumbar spinal cord, sequela | Lumbar |
| ICD-10-CM | S34.103A | S34103A | S34.1* | Unspecified injury to L3 level of lumbar spinal cord, initial encounter | Lumbar |
| ICD-10-CM | S34.103D | S34103D | S34.1* | Unspecified injury to L3 level of lumbar spinal cord, subsequent encounter | Lumbar |
| ICD-10-CM | S34.103S | S34103S | S34.1* | Unspecified injury to L3 level of lumbar spinal cord, sequela | Lumbar |
| ICD-10-CM | S34.104A | S34104A | S34.1* | Unspecified injury to L4 level of lumbar spinal cord, initial encounter | Lumbar |
| ICD-10-CM | S34.104D | S34104D | S34.1* | Unspecified injury to L4 level of lumbar spinal cord, subsequent encounter | Lumbar |
| ICD-10-CM | S34.104S | S34104S | S34.1* | Unspecified injury to L4 level of lumbar spinal cord, sequela | Lumbar |
| ICD-10-CM | S34.105A | S34105A | S34.1* | Unspecified injury to L5 level of lumbar spinal cord, initial encounter | Lumbar |
| ICD-10-CM | S34.105D | S34105D | S34.1* | Unspecified injury to L5 level of lumbar spinal cord, subsequent encounter | Lumbar |
| ICD-10-CM | S34.105S | S34105S | S34.1* | Unspecified injury to L5 level of lumbar spinal cord, sequela | Lumbar |
| ICD-10-CM | S34.109A | S34109A | S34.1* | Unspecified injury to unspecified level of lumbar spinal cord, initial encounter | Lumbar |
| ICD-10-CM | S34.109D | S34109D | S34.1* | Unspecified injury to unspecified level of lumbar spinal cord, subsequent encounter | Lumbar |
| ICD-10-CM | S34.109S | S34109S | S34.1* | Unspecified injury to unspecified level of lumbar spinal cord, sequela | Lumbar |
| ICD-10-CM | S34.111A | S34111A | S34.1* | Complete lesion of L1 level of lumbar spinal cord, initial encounter | Lumbar |
| ICD-10-CM | S34.111D | S34111D | S34.1* | Complete lesion of L1 level of lumbar spinal cord, subsequent encounter | Lumbar |
| ICD-10-CM | S34.111S | S34111S | S34.1* | Complete lesion of L1 level of lumbar spinal cord, sequela | Lumbar |
| ICD-10-CM | S34.112A | S34112A | S34.1* | Complete lesion of L2 level of lumbar spinal cord, initial encounter | Lumbar |
| ICD-10-CM | S34.112D | S34112D | S34.1* | Complete lesion of L2 level of lumbar spinal cord, subsequent encounter | Lumbar |
| ICD-10-CM | S34.112S | S34112S | S34.1* | Complete lesion of L2 level of lumbar spinal cord, sequela | Lumbar |
| ICD-10-CM | S34.113A | S34113A | S34.1* | Complete lesion of L3 level of lumbar spinal cord, initial encounter | Lumbar |
| ICD-10-CM | S34.113D | S34113D | S34.1* | Complete lesion of L3 level of lumbar spinal cord, subsequent encounter | Lumbar |
| ICD-10-CM | S34.113S | S34113S | S34.1* | Complete lesion of L3 level of lumbar spinal cord, sequela | Lumbar |
| ICD-10-CM | S34.114A | S34114A | S34.1* | Complete lesion of L4 level of lumbar spinal cord, initial encounter | Lumbar |
| ICD-10-CM | S34.114D | S34114D | S34.1* | Complete lesion of L4 level of lumbar spinal cord, subsequent encounter | Lumbar |
| ICD-10-CM | S34.114S | S34114S | S34.1* | Complete lesion of L4 level of lumbar spinal cord, sequela | Lumbar |
| ICD-10-CM | S34.115A | S34115A | S34.1* | Complete lesion of L5 level of lumbar spinal cord, initial encounter | Lumbar |
| ICD-10-CM | S34.115D | S34115D | S34.1* | Complete lesion of L5 level of lumbar spinal cord, subsequent encounter | Lumbar |
| ICD-10-CM | S34.115S | S34115S | S34.1* | Complete lesion of L5 level of lumbar spinal cord, sequela | Lumbar |
| ICD-10-CM | S34.119A | S34119A | S34.1* | Complete lesion of unspecified level of lumbar spinal cord, initial encounter | Lumbar |
| ICD-10-CM | S34.119D | S34119D | S34.1* | Complete lesion of unspecified level of lumbar spinal cord, subsequent encounter | Lumbar |
| ICD-10-CM | S34.119S | S34119S | S34.1* | Complete lesion of unspecified level of lumbar spinal cord, sequela | Lumbar |
| ICD-10-CM | S34.121A | S34121A | S34.1* | Incomplete lesion of L1 level of lumbar spinal cord, initial encounter | Lumbar |
| ICD-10-CM | S34.121D | S34121D | S34.1* | Incomplete lesion of L1 level of lumbar spinal cord, subsequent encounter | Lumbar |
| ICD-10-CM | S34.121S | S34121S | S34.1* | Incomplete lesion of L1 level of lumbar spinal cord, sequela | Lumbar |
| ICD-10-CM | S34.122A | S34122A | S34.1* | Incomplete lesion of L2 level of lumbar spinal cord, initial encounter | Lumbar |
| ICD-10-CM | S34.122D | S34122D | S34.1* | Incomplete lesion of L2 level of lumbar spinal cord, subsequent encounter | Lumbar |
| ICD-10-CM | S34.122S | S34122S | S34.1* | Incomplete lesion of L2 level of lumbar spinal cord, sequela | Lumbar |
| ICD-10-CM | S34.123A | S34123A | S34.1* | Incomplete lesion of L3 level of lumbar spinal cord, initial encounter | Lumbar |
| ICD-10-CM | S34.123D | S34123D | S34.1* | Incomplete lesion of L3 level of lumbar spinal cord, subsequent encounter | Lumbar |
| ICD-10-CM | S34.123S | S34123S | S34.1* | Incomplete lesion of L3 level of lumbar spinal cord, sequela | Lumbar |
| ICD-10-CM | S34.124A | S34124A | S34.1* | Incomplete lesion of L4 level of lumbar spinal cord, initial encounter | Lumbar |
| ICD-10-CM | S34.124D | S34124D | S34.1* | Incomplete lesion of L4 level of lumbar spinal cord, subsequent encounter | Lumbar |
| ICD-10-CM | S34.124S | S34124S | S34.1* | Incomplete lesion of L4 level of lumbar spinal cord, sequela | Lumbar |
| ICD-10-CM | S34.125A | S34125A | S34.1* | Incomplete lesion of L5 level of lumbar spinal cord, initial encounter | Lumbar |
| ICD-10-CM | S34.125D | S34125D | S34.1* | Incomplete lesion of L5 level of lumbar spinal cord, subsequent encounter | Lumbar |
| ICD-10-CM | S34.125S | S34125S | S34.1* | Incomplete lesion of L5 level of lumbar spinal cord, sequela | Lumbar |
| ICD-10-CM | S34.129A | S34129A | S34.1* | Incomplete lesion of unspecified level of lumbar spinal cord, initial encounter | Lumbar |
| ICD-10-CM | S34.129D | S34129D | S34.1* | Incomplete lesion of unspecified level of lumbar spinal cord, subsequent encounter | Lumbar |
| ICD-10-CM | S34.129S | S34129S | S34.1* | Incomplete lesion of unspecified level of lumbar spinal cord, sequela | Lumbar |
| ICD-10-CM | S34.131A | S34131A | S34.1* | Complete lesion of sacral spinal cord, initial encounter | Sacral/coccygeal |
| ICD-10-CM | S34.131D | S34131D | S34.1* | Complete lesion of sacral spinal cord, subsequent encounter | Sacral/coccygeal |
| ICD-10-CM | S34.131S | S34131S | S34.1* | Complete lesion of sacral spinal cord, sequela | Sacral/coccygeal |
| ICD-10-CM | S34.132A | S34132A | S34.1* | Incomplete lesion of sacral spinal cord, initial encounter | Sacral/coccygeal |
| ICD-10-CM | S34.132D | S34132D | S34.1* | Incomplete lesion of sacral spinal cord, subsequent encounter | Sacral/coccygeal |
| ICD-10-CM | S34.132S | S34132S | S34.1* | Incomplete lesion of sacral spinal cord, sequela | Sacral/coccygeal |
| ICD-10-CM | S34.139A | S34139A | S34.1* | Unspecified injury to sacral spinal cord, initial encounter | Sacral/coccygeal |
| ICD-10-CM | S34.139D | S34139D | S34.1* | Unspecified injury to sacral spinal cord, subsequent encounter | Sacral/coccygeal |
| ICD-10-CM | S34.139S | S34139S | S34.1* | Unspecified injury to sacral spinal cord, sequela | Sacral/coccygeal |
| ICD-10-CM | S34.3XXA | S343XXA | S34.3* | Injury of cauda equina, initial encounter | Cauda equina |
| ICD-10-CM | S34.3XXD | S343XXD | S34.3* | Injury of cauda equina, subsequent encounter | Cauda equina |
| ICD-10-CM | S34.3XXS | S343XXS | S34.3* | Injury of cauda equina, sequela | Cauda equina |
