# Supplementary material for: Machine learning–driven prediction of in-hospital cardiac arrest among critically ill patients with traumatic spinal cord injury: a multicenter cohort study
Source: Front Cardiovasc Med. 2026 Sep 14;13:1818433. doi: 10.3389/fcvm.2026.1818433 (PMC13617107; doi:10.3389/fcvm.2026.1818433)
Supplement: Supplementary file 4 [file Table2.docx]

| Supplementary Table 2 Demographic data and baseline characteristics of the training set | | | |
| --- | --- | --- | --- |
| Variable | Non-IHCA Group (N=655) | IHCA Group (N=43) | P-value |
| Age **[yr, median (IQR)]** | 59.00 (28.92) | 66.02 (21.00) | 0.011* |
| Height **[cm, median (IQR)]** | 173.00 (14.90) | 173.00 (11.30) | 0.422 |
| Weight **[Kg, median (IQR)]** | 81.60 (27.27) | 83.00 (14.88) | 0.531 |
| **Gender [N (%)]** |  |  |  |
| Female | 469 (71.6%) | 30 (69.8%) | 0.796 |
| Male | 186 (28.4%) | 13 (30.2%) |  |
| **Race [N (%)]** |  |  |  |
| White | 445 (67.9%) | 22 (51.2%) | 0.022* |
| Black | 61 (9.3%) | 3 (7.0%) |  |
| Other | 149 (22.7%) | 18 (41.9%) |  |
| **Rating System [median (IQR)]** | | | |
| CCI | 2.00 (3.00) | 4.00 (2.50) | 0.013* |
| GCS | 14.00 (2.00) | 15.00 (5.50) | 0.441 |
| SOFA | 2.00 (4.00) | 7.00 (4.00) | <0.001* |
| APACHE II | 14.00 (10.00) | 22.00 (13.50) | <0.001* |
| **Spinal Injury Level [N (%)]** | | | |
| Upper Cervical | 132 (20.2%) | 31 (72.1%) | <0.001* |
| Lower Cervical | 113 (17.3%) | 7 (16.3%) |  |
| Cervical (Unspecified) | 99 (15.1%) | 1 (2.3%) |  |
| Thoracic | 103 (15.7%) | 0 (0.0%) |  |
| Lumbar | 40 (6.1%) | 0 (0.0%) |  |
| Uncertain | 168 (25.6%) | 4 (9.3%) |  |
| **Associated Fracture [N (%)]** | | | |
| Fracture | 264 (40.3%) | 20 (46.5%) | 0.422 |
| Non-Fracture | 391 (59.7%) | 23 (53.5%) |  |
| **Injury Mechanism [N (%)]** | | | |
| Fall (High) | 166 (25.3%) | 16 (37.2%) | <0.001* |
| Fall (Low) | 117 (17.9%) | 5 (11.6%) |  |
| Motor Vehicle | 125 (19.1%) | 17 (39.5%) |  |
| Violence | 139 (21.2%) | 2 (4.7%) |  |
| Unknown | 108 (16.5%) | 3 (7.0%) |  |
| **Paralysis Type [N (%)]** | | | |
| Quadriplegia | 102 (15.6%) | 6 (14.0%) | <0.001* |
| Paraplegia | 85 (13.0%) | 15 (34.9%) |  |
| None/Uncertain | 468 (71.5%) | 22 (51.2%) |  |
| **Vasopressor [N (%)]** | | | |
| Non-Use | 512 (78.2%) | 19 (44.2%) | <0.001* |
| Use | 143 (21.8%) | 24 (55.8%) |  |
| **Mechanical Ventilation [N (%)]** | | |  |
| Non-Use | 479 (73.1%) | 20 (46.5%) | <0.001* |
| Use | 176 (26.9%) | 23 (53.5%) |  |
| **Vital Signs [median (IQR)]** | | | |
| SBP (mmHg) | 125.23 (22.38) | 118.61 (22.70) | 0.014* |
| DBP (mmHg) | 66.73 (13.07) | 64.11 (11.07) | 0.003* |
| MAP (mmHg) | 85.50 (13.89) | 81.67 (10.97) | 0.003* |
| Heart Rate (beats/min) | 63.00 (19.00) | 52.00 (21.50) | <0.001* |
| Resp Rate (breaths/min) | 17.56 (3.98) | 19.06 (4.90) | <0.001* |
| Temperature (­°C) | 36.30 (0.70) | 35.70 (2.27) | <0.001* |
| **Blood Gas [median (IQR)]** | | | |
| SpO_2_ (%) | 92.00 (6.00) | 92.00 (11.50) | 0.127 |
| Bicarbonate (mmol/L) | 22.00 (5.00) | 19.00 (4.00) | <0.001* |
| **Laboratory Tests [median (IQR)]** | | | |
| Hematocrit (%) | 38.00 (7.35) | 38.20 (9.85) | 0.932 |
| Hemoglobin (g/dL) | 10.50 (3.50) | 10.70 (2.95) | 0.802 |
| Platelets (10^9^/L) | 173.00 (89.50) | 152.00 (80.00) | 0.066 |
| WBC (10^9^/L) | 13.60 (7.92) | 16.50 (7.55) | 0.016* |
| Calcium (mmol/L) | 8.00 (1.05) | 7.70 (0.75) | 0.005* |
| Chloride (mmol/L) | 107.00 (6.00) | 109.00 (8.50) | 0.015* |
| Potassium (mmol/L) | 3.70 (0.70) | 3.00 (0.90) | <0.001* |
| Sodium (mmol/L) | 141.00 (4.00) | 142.00 (6.50) | 0.039* |
| Glucose (mg/dL) | 152.00 (64.50) | 179.00 (96.50) | <0.001* |
| Creatinine (mg/dL) | 0.90 (0.42) | 1.10 (0.70) | 0.025* |
| BUN (mg/dL) | 19.00 (12.00) | 20.00 (14.50) | 0.300 |
