# Supplementary material for: Machine learning–driven prediction of in-hospital cardiac arrest among critically ill patients with traumatic spinal cord injury: a multicenter cohort study
Source: Front Cardiovasc Med. 2026 Sep 14;13:1818433. doi: 10.3389/fcvm.2026.1818433 (PMC13617107; doi:10.3389/fcvm.2026.1818433)
Supplement: Supplementary file 5 [file Table3.docx]

| Supplementary Table 3 Demographic data and baseline characteristics of the external validation cohort | | | |
| --- | --- | --- | --- |
| Variable | Non-IHCA Group (N=507) | IHCA Group (N=51) | P-value |
| Age [yr, median (IQR)] | 58.75 (29.88) | 61.20 (30.39) | 0.625 |
| **Gender [N (%)]** | | | |
| Male | 343 (67.7%) | 35 (68.6%) | 1.000 |
| Female | 164 (32.3%) | 16 (31.4%) |  |
| **Spinal Injury Level [N (%)]** | | | |
| Upper Cervical | 113 (22.3%) | 25 (49.0%) | 0.001* |
| Lower Cervical | 95 (18.7%) | 10 (19.7%) |  |
| Cervical (Unspecified) | 62 (12.2%) | 7 (13.7%) |  |
| Thoracic | 82 (16.2%) | 2 (3.9%) |  |
| Lumbar | 24 (4.7%) | 2 (3.9%) |  |
| Uncertain | 131 (25.9%) | 5 (9.8%) |  |
| **Injury Mechanism [N (%)]** | | | |
| Fall (High) | 145 (28.6%) | 20 (39.2%) | 0.005* |
| Fall (Low) | 85 (16.8%) | 3 (5.9%) |  |
| Motor Vehicle | 89 (17.6%) | 17 (33.4%) |  |
| Violence | 95 (18.7%) | 4 (7.8%) |  |
| Unknown | 93 (18.3%) | 7 (13.7%) |  |
| **Paralysis Type [N (%)]** | | | |
| Quadriplegia | 80 (15.8%) | 17 (33.3%) | <0.001* |
| Paraplegia | 55 (10.8%) | 16 (31.4%) |  |
| None/Uncertain | 372 (73.4%) | 18 (35.3%) |  |
| **Vasopressor [N (%)]** | | | |
| Non-Use | 381 (75.1%) | 22 (43.1%) | <0.001* |
| Use | 126 (24.9%) | 29 (56.9%) |  |
| **Continuous Variables** | | | |
| MAP (mmHg) | 101.00 (23.00) | 80.00 (16.00) | <0.001* |
| Temperature (­°C) | 36.50 (0.60) | 35.50 (2.15) | <0.001* |
| Potassium (mmol/L) | 4.31 (0.70) | 3.57 (0.84) | <0.001* |
| Sodium (mmol/L) | 140.00 (5.00) | 137.00 (7.10) | 0.002* |
| Bicarbonate (mmol/L) | 22.00 (4.00) | 19.70 (5.40) | <0.001* |
| APACHE II | 14.00 (11.00) | 21.00 (9.00) | <0.001* |

* means significant
