# Supplementary material for: Machine learning–driven prediction of in-hospital cardiac arrest among critically ill patients with traumatic spinal cord injury: a multicenter cohort study
Source: Front Cardiovasc Med. 2026 Sep 14;13:1818433. doi: 10.3389/fcvm.2026.1818433 (PMC13617107; doi:10.3389/fcvm.2026.1818433)
Supplement: Supplementary file 6 [file Table4.docx]

| Supplementary Table 4 Comparison of basic cohort characteristics and final predictor distributions between the development and external validation cohorts | | | |
| --- | --- | --- | --- |
| Variable | Development Cohort (N=996) | External Cohort (N=558) | SMD |
| Age [yr, median (IQR)] | 58.00 (30.00) | 59.00 (30.19) | 0.010 |
| **Gender [N (%)]** | | | |
| Male | 716 (71.9%) | 378 (67.7%) | 0.090 |
| Female | 280 (28.1%) | 180 (32.3%) |  |
| **Spinal Injury Level [N (%)]** | | | |
| Upper Cervical | 219 (22.0%) | 138 (24.7%) | 0.156 |
| Lower Cervical | 153 (15.4%) | 105 (18.8%) |  |
| Cervical (Unspecified) | 159 (16.0%) | 69 (12.4%) |  |
| Thoracic | 141 (14.2%) | 84 (15.1%) |  |
| Lumbar | 55 (5.5%) | 26 (4.7%) |  |
| Uncertain | 269 (27.0%) | 136 (24.4%) |  |
| **Injury Mechanism [N (%)]** | | | |
| Fall (High) | 267 (26.8%) | 165 (29.6%) | 0.137 |
| Fall (Low) | 144 (14.5%) | 88 (15.8%) |  |
| Motor Vehicle | 168 (16.9%) | 106 (19.0%) |  |
| Violence | 224 (22.5%) | 99 (17.7%) |  |
| Unknown | 193 (19.4%) | 100 (17.9%) |  |
| **Paralysis Type [N (%)]** | | | |
| Quadriplegia | 134 (13.5%) | 97 (17.4%) | 0.110 |
| Paraplegia | 128 (12.9%) | 71 (12.7%) |  |
| None/Uncertain | 734 (73.7%) | 390 (69.9%) |  |
| **Vasopressor [N (%)]** | | | |
| Non-Use | 754 (75.7%) | 403 (72.2%) | 0.079 |
| Use | 242 (24.3%) | 155 (27.8%) |  |
| **Continuous Variables** | | | |
| MAP (mmHg) | 85.45 (14.23) | 99.50 (23.00) | 0.994* |
| Temperature (­°C) | 36.29 (0.76) | 36.50 (0.56) | 0.184 |
| Potassium (mmol/L) | 3.60 (0.70) | 4.30 (0.73) | 1.079* |
| Sodium (mmol/L) | 141.00 (5.00) | 139.95 (5.07) | 0.132 |
| Bicarbonate (mmol/L) | 22.00 (5.00) | 22.00 (4.00) | 0.036 |
| APACHE II | 15.00 (12.00) | 15.00 (12.00) | 0.004 |

*Indicates substantial imbalance in variable distributions between the two cohorts, defined as an absolute SMD greater than 0.25.
