# Supplementary material for: Machine learning–driven prediction of in-hospital cardiac arrest among critically ill patients with traumatic spinal cord injury: a multicenter cohort study
Source: Front Cardiovasc Med. 2026 Sep 14;13:1818433. doi: 10.3389/fcvm.2026.1818433 (PMC13617107; doi:10.3389/fcvm.2026.1818433)
Supplement: Supplementary file 7 [file Table5.docx]

| Supplementary Table 5 Apparent and optimism-corrected performance of candidate models in the training set | | | | |
| --- | --- | --- | --- | --- |
| Model | Apparent AUC | Optimism-corrected AUC | Apparent AP | Optimism-corrected AP |
| AdaBoost | 0.832 | 0.812 | 0.543 | 0.519 |
| CatBoost | 0.817 | 0.801 | 0.632 | 0.603 |
| DT | 0.689 | 0.655 | 0.482 | 0.471 |
| GBM | 0.811 | 0.795 | 0.619 | 0.594 |
| KNN | 0.770 | 0.755 | 0.556 | 0.535 |
| LightGBM | 0.833 | 0.812 | 0.572 | 0.544 |
| LR | 0.874 | 0.854 | 0.719 | 0.709 |
| MLP | 0.639 | 0.617 | 0.462 | 0.437 |
| RF | 0.787 | 0.759 | 0.625 | 0.611 |
| SVM | 0.862 | 0.848 | 0.737 | 0.709 |
| XGBoost | 0.822 | 0.804 | 0.559 | 0.532 |
| Stacking model | 0.953 | 0.934 | 0.848 | 0.823 |

AUC, area under the receiver operating characteristic curve; AP, average precision.
