# Supplementary material for: Machine learning–driven prediction of in-hospital cardiac arrest among critically ill patients with traumatic spinal cord injury: a multicenter cohort study
Source: Front Cardiovasc Med. 2026 Sep 14;13:1818433. doi: 10.3389/fcvm.2026.1818433 (PMC13617107; doi:10.3389/fcvm.2026.1818433)
Supplement: Supplementary file 8 [file Table6.docx]

| Supplementary Table 6 Calibration performance of machine-learning models in the training, internal validation, and external validation sets | | | | | |
| --- | --- | --- | --- | --- | --- |
| Set | Model | CITL | Calibration slope | Brier score | ECE |
| Training set | Stacking model | -0.237 (-0.520, -0.053) | 1.171 (0.923, 1.418) | 0.020 (0.013, 0.028) | 0.027 (0.023, 0.040) |
|  | SVM | -1.661 (-2.041, -1.280) | 0.576 (0.416, 0.735) | 0.080 (0.067, 0.094) | 0.089 (0.075, 0.113) |
|  | XGBoost | -1.635 (-2.037, -1.232) | 0.448 (0.322, 0.573) | 0.082 (0.067, 0.097) | 0.096 (0.079, 0.117) |
|  | RF | -1.364 (-1.704, -1.025) | 0.525 (0.330, 0.720) | 0.086 (0.073, 0.099) | 0.094 (0.081, 0.118) |
|  | LR | -2.141 (-2.539, -1.743) | 0.608 (0.435, 0.782) | 0.086 (0.073, 0.099) | 0.131 (0.114, 0.151) |
|  | GBM | -1.933 (-2.368, -1.498) | 0.378 (0.275, 0.482) | 0.089 (0.073, 0.107) | 0.102 (0.085, 0.125) |
|  | CatBoost | -1.722 (-2.197, -1.248) | 0.291 (0.208, 0.375) | 0.089 (0.072, 0.108) | 0.092 (0.076, 0.115) |
|  | KNN | -1.308 (-1.908, -0.811) | 0.062 (0.040, 0.083) | 0.096 (0.081, 0.112) | 0.105 (0.088, 0.128) |
|  | LightGBM | -3.085 (-3.718, -2.453) | 0.159 (0.115, 0.204) | 0.100 (0.081, 0.119) | 0.108 (0.089, 0.131) |
|  | DT | -2.117 (-2.918, -1.496) | 0.039 (0.020, 0.058) | 0.111 (0.093, 0.130) | 0.130 (0.111, 0.155) |
|  | MLP | -2.284 (-2.601, -1.967) | 0.421 (0.033, 0.808) | 0.159 (0.150, 0.169) | 0.291 (0.270, 0.312) |
|  | AdaBoost | -2.313 (-2.623, -2.003) | 3.052 (2.104, 3.999) | 0.160 (0.154, 0.166) | 0.324 (0.306, 0.342) |
| Internal validation set | Stacking model | -0.312 (-0.847, -0.177) | 1.333 (0.773, 1.893) | 0.013 (0.005, 0.023) | 0.033 (0.026, 0.047) |
|  | RF | -2.180 (-2.754, -1.606) | 0.400 (0.072, 0.728) | 0.112 (0.092, 0.134) | 0.167 (0.139, 0.203) |
|  | SVM | -2.347 (-2.970, -1.724) | 0.293 (0.060, 0.527) | 0.115 (0.090, 0.142) | 0.151 (0.124, 0.191) |
|  | XGBoost | -2.515 (-3.154, -1.877) | 0.317 (0.107, 0.526) | 0.119 (0.094, 0.147) | 0.155 (0.129, 0.196) |
|  | CatBoost | -3.069 (-3.825, -2.314) | 0.200 (0.052, 0.347) | 0.125 (0.095, 0.158) | 0.147 (0.119, 0.190) |
|  | LR | -2.581 (-3.213, -1.950) | 0.238 (0.040, 0.436) | 0.126 (0.102, 0.152) | 0.194 (0.165, 0.231) |
|  | DT | -2.572 (-3.183, -1.961) | 0.088 (-0.005, 0.181) | 0.130 (0.105, 0.156) | 0.200 (0.166, 0.233) |
|  | GBM | -2.689 (-3.345, -2.033) | 0.252 (0.070, 0.434) | 0.130 (0.102, 0.160) | 0.158 (0.134, 0.203) |
|  | KNN | <-3 | 0.020 (-0.007, 0.048) | 0.141 (0.112, 0.172) | 0.182 (0.147, 0.220) |
|  | LightGBM | -5.144 (-6.078, -4.211) | 0.096 (0.026, 0.166) | 0.144 (0.108, 0.183) | 0.161 (0.124, 0.202) |
|  | MLP | -2.702 (-3.256, -2.148) | -0.085 (-0.585, 0.415) | 0.172 (0.152, 0.193) | 0.310 (0.283, 0.344) |
|  | AdaBoost | -2.705 (-3.246, -2.163) | 1.241 (0.322, 2.161) | 0.174 (0.162, 0.187) | 0.352 (0.327, 0.378) |
| External validation set | Stacking model | 0.541 (-0.079, 1.161) | 1.318 (0.943, 1.693) | 0.013 (0.008, 0.020) | 0.020 (0.016, 0.032) |

Values are estimates (95% CIs). CITL, calibration-in-the-large; ECE, expected calibration error.
