# Supplementary material for: Machine learning–driven prediction of in-hospital cardiac arrest among critically ill patients with traumatic spinal cord injury: a multicenter cohort study
Source: Front Cardiovasc Med. 2026 Sep 14;13:1818433. doi: 10.3389/fcvm.2026.1818433 (PMC13617107; doi:10.3389/fcvm.2026.1818433)
Supplement: Supplementary file 9 [file Table7.docx]

| Supplementary Table 7 Hyperparameters of each machine learning model | |
| --- | --- |
| Models | Hyperparameters |
| AdaBoost | best_params_adaboost = {"clf__n_estimators":400,"clf__learning_rate":0.5,"clf__algorithm":"SAMME","clf__estimator__max_depth":2,"clf__estimator__min_samples_split":10,"clf__estimator__min_samples_leaf":2,"clf__estimator__ccp_alpha":0.0,"clf__estimator__class_weight":"balanced"} |
| CatBoost | best_params_catboost={"clf__depth":6,"clf__iterations":800,"clf__learning_rate":0.1,"clf__l2_leaf_reg":6.0,"clf__loss_function":"Logloss","clf__eval_metric":"AUC","clf__scale_pos_weight":15,"clf__bootstrap_type":"Bernoulli","clf__subsample":0.8,"clf__random_state":42} |
| DT | best_params_dt={"clf__max_depth":5,"clf__min_samples_leaf":5,"clf__min_samples_split":10,"clf__criterion":"gini","clf__class_weight":"balanced","clf__random_state":42} |
| GBM | best_params_gbm={"clf__learning_rate":0.1,"clf__n_estimators":300,"clf__max_depth":3,"clf__min_samples_leaf":5,"clf__min_samples_split":10,"clf__subsample":0.8,"clf__random_state":42} |
| KNN | best_params_knn={"clf__n_neighbors":11,"clf__p":1,"clf__weights":"distance","clf__metric":"minkowski","clf__algorithm":"auto"} |
| LightGBM | best_params_lgbm={"clf__learning_rate":0.1,"clf__max_depth":-1,"clf__min_child_samples":20,"clf__num_leaves":31,"clf__reg_lambda":1.0,"clf__n_estimators":500,"clf__subsample":0.8,"clf__colsample_bytree":0.8,"clf__scale_pos_weight":15,"clf__random_state":42} |
| Logistic | best_params_lr={"clf__penalty":"l2","clf__C":1.0,"clf__solver":"liblinear","clf__class_weight":"balanced","clf__max_iter":1000,"clf__random_state":42} |
| MLP | best_params_mlp={"clf__hidden_layer_sizes":(64,32),"clf__alpha":0.0001,"clf__learning_rate_init":0.001,"clf__solver":"adam","clf__activation":"relu","clf__batch_size":"auto","clf__max_iter":500,"clf__early_stopping":True,"clf__random_state":42} |
| RF | best_params_rf={"clf__n_estimators":300,"clf__max_depth":None,"clf__min_samples_leaf":1,"clf__min_samples_split":2,"clf__max_features":"sqrt","clf__bootstrap":True,"clf__class_weight":"balanced","clf__random_state":42} |
| SVM | best_params_svm={"clf__C":10,"clf__kernel":"rbf","clf__gamma":"scale","clf__class_weight":"balanced","clf__probability":True,"clf__random_state":42} |
| XGBoost | best_params_xgb={"clf__learning_rate":0.05,"clf__max_depth":3,"clf__subsample":0.7,"clf__colsample_bytree":0.7,"clf__reg_lambda":1.0,"clf__n_estimators":500,"clf__min_child_weight":1,"clf__gamma":0,"clf__scale_pos_weight":15,"clf__objective":"binary:logistic","clf__eval_metric":"auc","clf__random_state":42} |
| Stacking (Meta-learner) | final_best_params_level2={"meta__penalty":"l2","meta__C":1.0,"meta__solver":"liblinear","meta__class_weight":"balanced","meta__max_iter":1000,"meta__random_state":42} |
